# A robust and personalized privacy-preserving approach for adaptive clustered federated distillation

**DOI:** 10.1038/s41598-025-96468-8

**Published:** 2025-04-23

**Authors:** Mai Shawkat, Zainab H. Ali, Mofreh Salem, Ali El-desoky

**Affiliations:** 1 Department of Electronics and Communications Engineering, Mansoura Higher Institute for Engineering and Technology, Mansoura, Egypt; 2 Department of Embedded Network Systems and Technology, Faculty of Artificial Intelligence, Kafrelsheikh University, El-Geish St, Kafrelsheikh, 33516 Egypt; 3 Department of Electronics and Computer Engineering, School of Engineering and Applied Sciences, Nile University, Giza, Egypt; 4 Department of Computer Engineering and Systems, Faculty of Engineering, Mansoura University, Mansoura, Egypt

**Keywords:** Deep learning, Federated learning, Knowledge distillation, Meta learning, Privacy protection, Engineering, Electrical and electronic engineering

## Abstract

Federated learning (FL) is a promising approach that addresses privacy, and scalability concerns in contrast to traditional centralized methods. Challenges such as personalization and data heterogeneity issues remain critical. Clustered federated learning (CFL) has been proposed as a promising approach to alleviate these issues by establishing specialized global models for sets of similar users. Although CFL enhances adaptability to highly statistically heterogeneous environments, it may suffer from real-time distribution changes due to limitations in fixed cluster configurations. This study presents the robust model of personalized federated distillation (RMPFD), a personalized and privacy-enhanced framework. The RMPFD framework employs an adaptive hierarchical clustering strategy to generate semi-global models by grouping clients with similar data distributions, allowing them to train independently. Meta-learning is used in each cluster to enhance the personalization of the local models and the classification accuracy of the non-independent and Identically distributed (non-IID) data distributions. Experimental evaluations conducted on CIFAR- 10, CIFAR- 100, Fashion-MNIST and Enron email datasets reveal that RMPFD reduces communication overhead by approximately 15% and 20%, compared to Federated Averaging (FedAvg) and other baseline techniques. Moreover, the RMPFD framework improves the convergence rates and classification accuracy, leading to an improvement of over 12% in performance compared to traditional FL methods.

## Introduction

To address the rising demand for machine learning (ML), organizations seek ways to enhance the implementation of the ML models through data sharing^1^. However, strict privacy regulations have led to the data silos phenomenon^2^. In response to this challenge, federated learning (FL) was developed as a promising solution, allowing for parameter exchange instead of direct data sharing^1–3^. In FL, each data provider collaborates with a central server to train a global model while ensuring the privacy of the original data. The methods used to implement FL differ according to the distinct requirements of the system.

Deep learning (DL) advancements rely on artificial neural networks, which can significantly improve numerous AI applications. It involves natural language processing (NLP), speech recognition, and object detection. These applications typically require large volumes of training data, often collected in ways that raise privacy concerns. The FL has gained attention as a distributed ML framework that facilitates collaborative training of a unified model across distinct datasets^2,4–6^. Local training conducted by individual clients can be limited due to insufficient data or low data quality, which can negatively influence the development of underperforming inference models.

In the current FL setup, a network of specific clients such as businesses, educational institutions, and edge devices collaborate to collectively train the global model supervised by the coordinator server. Client data is kept securely on local devices, and only the model parameters are exchanged to the server^7–9^. While FL offers significant potential benefits, there are persistent technical issues, particularly related to statistical data heterogeneity and system heterogeneity. These issues significantly hamper the learning process and increase complexity. System heterogeneity refers to variations in computational power or communication channels among clients. Several approaches, such as active sampling, have been proposed to tackle this problem^7,10–12^. Statistical heterogeneity is attributed to the discrete distribution of heterogeneous data in a federated system. A variety of methodologies have been suggested to tackle this issue^13^.

To examine model heterogeneity in FL, all data holders collaborate to train a single statistical model for numerous small-edge devices. However, in sensitive sectors such as healthcare, hospitals prefer to develop their unique models due to concerns regarding patient data privacy. The FedBKD framework was proposed^10^, allowing clients to autonomously craft their models by leveraging a simple mean aggregation. However, this can significantly impact system and data heterogeneity issues. This technique proposes a high entropy methodology for making comprehensive predictions by applying a weighted average to combine multiple model outcomes. The goal of increasing global prediction entropy is to gain additional information in soft label form during the collaborative learning rounds, leading to improved and stabilized model performance.

It is crucial to note that the increase in entropy of global predictions can be recognized because a model’s class scores for a single input encompass both accurate and inaccurate responses in the form of relative probabilities. While our primary goal is to obtain the correct answer, the incorrect responses can still provide valuable knowledge in the cases of small probabilities^14^. For example, different models demonstrate varying performance levels when tested on similar data samples. Certain models display varying levels of confidence, influenced by their architectural difficulty and unique data distributions. Nevertheless, even models with low confidence levels provide valuable knowledge. Deliberately increasing the entropy of overall predictions during the collaborative learning phase rounds results in a greater depth of knowledge, ultimately enhancing and stabilizing the performance of each model.

**Fig. 1:**
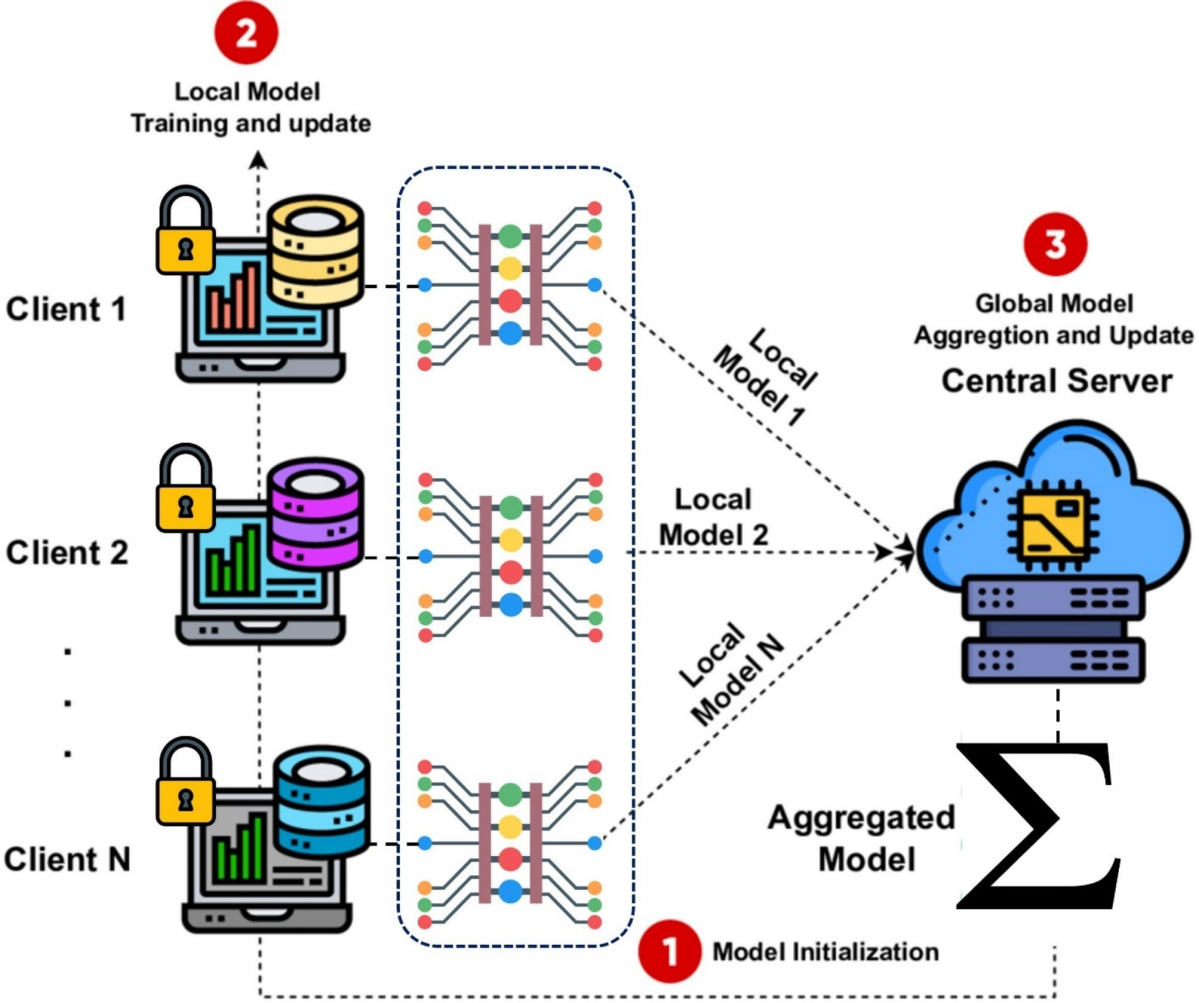
Overview of the federated system architecture in cloud-edge collaborative networks.

In the classic FL structure, all participating devices are trained using a single global model, as illustrated in Fig. 1. However, in different application settings, individual participants may need their separate personalized local models, which the current framework does not accommodate. For instance, deploying the FL framework on edge devices results in a lightweight global model. Other clients may need custom network architecture for a trained model to respond to local data. The paper focuses on cross-silo FL^15^, which typically involves a limited number of clients and adequate computing resources. Despite advancements in research, FL still faces significant challenges^16^. A prominent issue is model heterogeneity, which significantly impacts the effectiveness of FL implementations^17^.

Within standard FL, the global model is a conduit for knowledge dissemination, capturing valuable information and encoding data features from all local datasets. Additionally, the incorporation of knowledge distillation technology^18^ into DL can assist in training a compact global model from expert models. Consequently, the reciprocal nature of the knowledge transfer process enables the local model to compute the accuracy and loss functions, which are used to evaluate model performance.

This context introduces the Robust Model of Personalized Federated Distillation (RMPFD). This model enables clients to tailor their personalized models by integrating local training, global aggregation, and personalization techniques. Additionally, RMPFD supports efficient and privacy-preserving collaboration during the global model training, which acts as a conduit for knowledge transfer. This process facilitates mutual learning between global and local models, enhancing overall generalization performance. A notable advantage of RMPFD is its ability to mitigate the risks associated with gradient leakage^19^. In FL, each client transmits updates of the full model parameters to the server. If the malicious adversaries control the server, they may exploit gradient information to reconstruct local client data. Current solutions include differential privacy and cryptographic techniques^20^. Using cryptographic techniques, all client nodes can maintain privacy by encrypting inputs and transmitting encrypted results to the server. A secure cross-modal framework integrates collective matrix factorization with homomorphic encryption^18^ to construct an efficient and precise scheme for real-world IoT applications. This approach has drawbacks, such as being computationally intensive and slow. It involves adding differential privacy mechanisms to the local updates before model aggregation. However, this can sometimes reduce the utility of data, which may be unacceptable in certain scenarios. In RMPFD, all clients contribute to reconstructing local data from the model gradient. In comparison to differential privacy and homomorphic encryption, RMPFD provides a more favorable swap between computational cost and model performance.

The novelties of this study can be summarized as follows:

i. Introducing the RMPFD as a new personalized FL model incorporating a high-entropy aggregation method that balances personalization and generalization in federated settings by improving model robustness.
ii. Proposing an adaptive hierarchical clustering strategy that creates intermediate semi-global models. This strategy clusters clients with similar data distributions, allowing them to collaboratively train semi-personalized models, advancing model adaptability and speeds up convergence through non-IID data distributions.
iii. Exploring the benefits of the RMPFD multi-model in addressing gradient privacy issues by integrating knowledge distillation techniques to transfer historical personalized knowledge between global and local models. This technique helps mitigate statistical heterogeneity and reduces dependency on raw data exchange while maintaining model effectiveness.
iv. The framework incorporates meta-learning techniques within clusters to optimize personalization. This approach enhances the adaptability of client models to extreme data heterogeneity, improving their classification accuracy.
v. Conducting extensive evaluation on various model architectures and standard datasets, under highly heterogeneous data distributions. The experimental results demonstrate significant improvements in convergence speed. Lower data loss and high classification accuracy compared to baseline FL algorithms.

The following sections of this study cover, Section “Background and related work” presents an overview of FL concepts, accompanied by a thorough review and analysis of related works. Section “Proposed robust model of personalized federated distillation (RMPFD)” outlines the background of personalized federated meta-learning and provides detailed technical advancement of RMPFD. Section “Experimental work and discussion” shows the extensive experiments carried out to assess the performance of the RMPFD covering aspects such as comparative accuracy analysis, communication efficiency, sensitivity analysis, and various system performance metrics. Finally, Section “Conclusion and future work” summarizes the paper and proposes possible future avenues for the proposed framework.

## Background and related work

FL is an innovative decentralized machine learning framework that facilitates collaborative training among various artificial intelligence models using locally stored data, all while maintaining the integrity and privacy of the decentralized data^8^. This methodology is primarily designed to safeguard the privacy of sensitive information. In a conventional FL framework, edge devices collectively participate in the training of a global model by aggregating model parameters through techniques such as federated averaging (FedAVG)^21,22^ and standard gradient descent (SGD)^23^. Communication-Mitigated Federated Learning (CMFL)^24^ offers clients valuable insights into overarching trends in model updates by minimizing redundant data uploads. This approach improves communication efficiency and speeds up the convergence of the learning process. The federated dropout (FedDrop) scheme^25^ enhances the traditional dropout technique for model pruning. It effectively reduces communication overhead and the computational load on devices while also helping to mitigate overfitting. The Weighted Federated Communication (Weighted FedCOM) framework^26^ is a refined approach proposed to merge weighted aggregation with model compression. It allocates weights to client contributions relied on the accuracy of their local models, significantly reducing communication overhead. In traditional FL frameworks, clients receive the same local model in the training process, which may be a significant challenge related to statistical heterogeneity and personalization^16,27^ Personalized Federated Learning (PFL)^28,29^ was introduced to solve these issues, in which customized models are generated for discrete clients or specific groups of clients. Moreover, Clustered Federated Learning (CFL) techniques are used to categorize clients with similar data distribution into groups to produce adapted global models tailored to each group^30–32^. The FedFomo^33^ and FedPer^34^ methodologies aim to address the limitations of local models by prioritizing aggregated parameters and local adaptation. Traditional FL frameworks generally operate with a single global for all clients. This can work for IID data, but it often fails in practical applications characterized by diverse user behaviors and non-IID data distributions^35,36^. PFL techniques mitigate this discrepancy by customizing models to accommodate the specific requirements of individual clients or clusters.

Knowledge distillation is a process through which insufficient data models benefit from larger, more complex learning models, which plays a vital role in this context. Approaches such as Federated Model Distillation (FedMD)^37^ have been developed to enhance personalized learning while reducing computational and communication overheads. This framework employs transfer learning techniques derived from Federated Knowledge Distillation (FedKD), allowing edge devices to develop and refine personalized models by sharing model gradients, with a coordinating server supervising the process^38,39^. However, this approach may expose sensitive information, raising concerns regarding privacy and security. It has shown that malicious users can reconstruct original datasets and high-resolution images from shared gradients. Existing defense mechanisms against such attacks often exhibit drawbacks, including high computational costs and low efficiency^40^.

A ciphertext-based algorithm for user selection^41^ is presented in the framework of FL, specifically targeting users with extensive and uniformly distributed datasets. This effectively cleans out anomalous model parameters that significantly diverge from the global model, enhancing its robustness while ensuring local data privacy. In decentralized FL, malicious users pose a risk to both trust and model performance. The EVFL-DCs scheme^42^ enhances verifiability and privacy by introducing double commitments based on blockchain technology^43^. This mechanism ensures consistency between noisy gradients and their corresponding commitments, mitigating adversarial inconsistencies. Blockchain integration replaces the traditional central server, addressing the issue of a single point of failure. Moreover, the FL scheme within Digital Twin for Mobile Networks (DTMN) proposed in^44^. They have developed a behavior model to assess local and recommended trust, considering factors such as stability and reliability. It incorporates adaptive weight calculation to balance local models and recommended trust.

Despite the challenges encountered, convergence is typically achieved after several rounds of communication. The applications of PFL are diverse, ranging from healthcare where the protection of patient data is a critical priority to business settings that require tailored recommendations and the advancement of FL-based medical imaging applications^45^. The integration of clustered hierarchies within FL facilitates the development of intermediate global models closely aligned with the local distribution of clients. This approach enhances personalization and improves the convergence time by allowing selective model updates and aggregating distilled information.

### Personalized FL

In Personalized Federated Learning (PFL), clients refine local models by obtaining an improved model that aligns with their specific data distributions. Recent research has predominantly centered around the conventional approach to FL, which involves maintaining a singular global model. However, this approach is not suitable when a client requires a personalized learning model^46^. Numerous studies have focused on personalization in FL^28,29,32–36^. For instance, some researchers have suggested a CFL approach that involves classifying hierarchical clustering clients into different clusters based on the updated gradient similarity of the local model^47^. Applying transfer learning techniques across these clusters makes users benefit from models adapted to similar clients rather than the entire population, which increases model relevance and personalization potential. Furthermore, a strategy for combining local and global models was suggested, which included a penalty parameter for customization to boost the efficiency of the personalized model. A novel framework called HeteroFL has been introduced^48^, which involves multiple diverse devices training local models. In this framework, the aggregated models train locally by fine-tuning features belonging to the local majority class. These features are obtained by the global model utilizing a process of model parameter decoupling. The trained global and local models are then integrated through transfer learning to create an ultimate personalized model^29,39–42^. These studies have focused on optimizing personalized models by using meta-learning, regularization, and fine-tuning optimization strategies. Also, by employing techniques like transfer learning, Model-Agnostic Meta-Learning (MAML), and contextual bandits, PFL systems can more effectively meet diverse user needs while ensuring data privacy and efficiency.

However, relying solely on one unified global model among all devices may lead to a lack of diversity in generalization across various data distributions. Recent studies have explored utilizing numerous global models on the federated system sides to tackle this issue. For instance, CFL methods endeavor to group devices into clusters and create personalized global models for each cluster. FedAMP^35^ preserves personalized local models tailored for each client to promote constructive collaboration among clients with strong clustering characteristics. FedFomo^33^ encourages clients with similar data distributions to utilize mutual local validation data to analyze the higher personalized weights. However, these methods require additional validation data representing the target distribution, which can be impractical in some scenarios.

### Federated meta-learning

Effective DL models depend on their ability to extract feature representations, and pattern classifications from raw data using extensive parameters. However, this often demands substantial computational power and storage capacity. Implementing these advanced techniques on limited resources systems is a critical challenge that must be addressed^49^. One practical solution involves quantizing and compressing the DL model to reduce the parameter size, a technique known as a personalized and adaptive differentially private federated meta-learning (PADP-FedMeta)^50^. This method trains a personalized, compressed model for each client by aggregating knowledge and adaptive privacy parameters during each federation round, which enhances convergence speed, particularly when handling non-IID datasets. Model compression involves the fundamental information extraction from a complex, large-scale model to develop a more compact and efficient version while ensuring effectiveness. This is achieved in FedMeta by sharing a parameterized algorithm rather than utilizing a global model, as seen in prior methods of model compression methods, including knowledge distillation, parameter clipping, and quantization, which contribute to reducing the model’s size and improving its efficiency for deployment on edge devices^39^. Different studies have proposed various methods to optimize the distillation process.

For instance, certain studies have leveraged data from intermediate layers in conjunction with predictions from the final layer to facilitate the training of a compact model. Knowledge distillation has demonstrated effectiveness in model compression and knowledge transfer. Consequently, it has recently gained significant traction in PFL and federated meta-learning applications. However, there are a variety of FL frameworks proposed to tackle the challenge of diversity models by using knowledge distillation^10,14,51^ such as FedMD^37^. These approaches show great promise in enhancing the privacy of FL representations aiming to improve communication efficiency and manage data heterogeneity.

### Personalized FedKD

The Personalized Federated Knowledge Distillation (FedKD) has been introduced to address the model heterogeneity and communication overhead complexities^10^. This decentralized framework employs adaptive mutual distillation and dynamic gradient compression methods, enabling devices to customize their local mentor models while collaboratively training on their datasets. The diversity of local models in FedKD provides inherent protection against gradient leakage attacks by altering the training approach of the global model. The FedKD methodology employs a deep-to-shallow layer-dropping mechanism during the knowledge transfer process, which enhances the effectiveness of personalized models. Each participant possesses a unique model tailored to their private dataset, while a public dataset is assigned among all clients. The class notches calculated on the public dataset and sends the counts over the network to the server. Then, FedKD employs average class counts for knowledge transfer and communicates it back to the clients as the soft label of the public dataset. However, the production of a public dataset may compromise data confidentiality, rendering it impractical in various scenarios. Moreover, transfer learning^13^ and meta-learning^19,20,49^ are acknowledged as effective methods for tackling issues of model heterogeneity. The primary challenge lies in customizing the client model without compromising its performance and adaptability.

## Proposed robust model of personalized federated distillation (RMPFD)

**Fig. 2:**
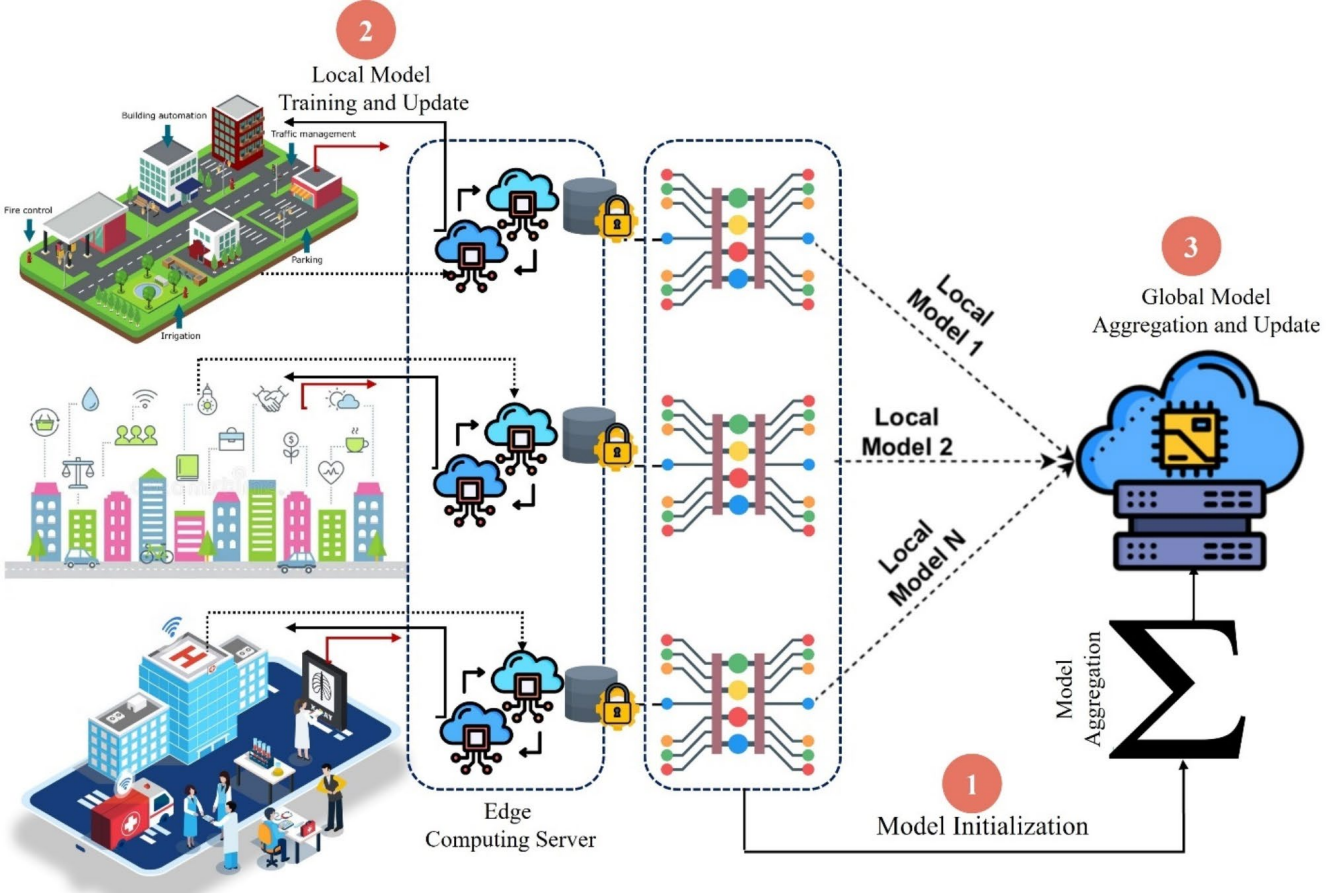
An Overview of RMPFD Framework.

The RMPFD approach provides a sophisticated framework for PFL, offering notable advantages in efficiency, adaptability, privacy-preserving, and resource management. By combining meta-learning and knowledge transfer, RMPFD facilitates rapid adaptation to non-IID data, effective knowledge transfer, and improved model generalization. This framework prioritizes the development of personalized models that can adapt seamlessly across various clients, to make federated systems more flexible. It highlights the importance of preserving personalized historical knowledge by utilizing a personalized model (PM) to supervise local training for each client. The local models represented as *θt*, are stored as PMs to support local training in subsequent communication rounds. Furthermore, the global model can be enhanced to increase generalization accuracy through methods such as momentum or clustering. Furthermore, FedMeta is employed in the recently updated project management system during the client updates phase to facilitate transfer knowledge from the previous model to the current model. The theoretical and experimental benefits of personalized FedMeta and FedKD are discussed in Sections “Federated meta-learning” and “Personalized FedKD”, respectively. This section presents an in-depth examination of the RMPFD framework and analyzes its characteristics in terms of privacy and heterogeneity. It also delves into the system design of the proposed framework. Figure 2 illustrates the core concept of RMPFD and the workflow of the RMPFD framework in the edge computing environment. The FL process follows a client-server framework, with edge devices acting as the clients. Table 1 determines the notations used in this study.

**Table 1:** Symbol explanations.

Symbol	Explanation
$\:{\theta\:}_{i}$	Personalized Model
i	Number of clients
*θ*	The global model
*θt*	The local model
$\:L\left(\theta\:\right)$	Cross entropy
*D*	Dataset $\:D={\left|\left({x}_{1},{y}_{1}\right)\right|}_{i=1}^{N}$
*N*	Number of samples
$\:{n}^{i}$	Private dataset belonging to the *i*th node
*M*	Maximum number of clusters
*m*	The label of the *m*th category
*B*	Batch size
$\underline{E}$	Epoch of local training
*C*	Portion of selected devices
$\:\eta\:$	Learning rate
$\:T$	Threshold for merging clusters
$\:CT$	Gradient Clipping Threshold
$\:{L}_{ML}$	Distillation loss
α	The learning rate
$\:f$	Probability distribution over classes
$\:{l}_{i}$	Logit value of the *i*th class
$\:{D}_{ML}$	Meta-learning knowledge distillation divergence
Ɲ(0, *σ*^2^)	Gaussian noise with mean 0 and variance $\:{\sigma\:}^{2}$

In a centralized learning environment, a personalized model is trained by using a *C*-class supervised classification task on a global model *θ*. The total number of participants connected to a server is denoted as *i*, with each client node having its local private dataset. The dataset *D* containing *N* samples belonging to class *x*,* y* as predicted by the global model$\:\:\theta\:$, is denoted as

$\:f\left({x}_{i},\theta\:\right)$. The cross-entropy error $\:L\left(\theta\:\right)\:$is estimated as the sum of the product of correct labels and the logarithm of predicted values over all samples and classes, using the following indicator function:

1 $$\:L\left(\theta\:\right)=\sum\:_{i=1}^{N}\:\sum\:_{==1}^{M}\:I\left({y}_{i},m\right)\text{l}\text{o}\_\text{s}\text{n}\text{i}\text{p}2\text{g}f\left({x}_{i},\theta\:\right)$$

Initialize the local model training by dividing dataset D into multiple batches, with *B* as local batch size. The model parameters are then updated using the SGD algorithm.

2 $$\:\theta\:\:\leftarrow\:\:\theta\:\:\--\:\eta\:\nabla\:L\left(\theta\:\right)$$

After completing multiple training cycles (epochs), the final model $\:{\theta\:}_{t+1}$ will be generated. In the traditional FL setting, the raw training data cannot be exchanged among various clients due to security threats and privacy issues. The raw training data can be transformed into model parameters to extract knowledge through learning and aggregating shared parameters to overcome this obstacle. The server distributes the global model $\:{\theta\:}_{t}\:$to clients, which then uses its local dataset $\:{D}_{i}$ to train $\:{\theta\:}_{t+1}^{i}$ (initialized by *θt*) and generates the local model. The mathematical equation indicates the global model can be extracted as follows:

3 $$\:{\theta\:}_{t+1}\leftarrow\:\sum\:_{i}\:\frac{{N}^{i}}{N}{\theta\:}_{t+1}^{i}$$

DL models leverage extensive data to extract meaningful features from raw data, necessitating substantial computing resources. Techniques such as quantization and compression can aid in reducing the parameter size. One popular compression method used in meta-learning is Knowledge Distillation^34^, which involves transferring learned knowledge by using the output of the last neural layer of the large model as a soft label. The soft label indicates the probability $\:f\left({l}_{i},T\right)$ of the sample being classified into each class as follows:

4 $$\:f\left({l}_{i},T\right)=\frac{exp\left({l}_{i}/T\right)}{\sum\:_{k}\:\:exp\left({l}_{k}/T\right)}$$

The distillation loss function effectively quantifies the disparity between the logits produced by the smaller model and those generated by the bigger model as follows:

5 $$\:{L}_{ML}={D}_{ML}\left(f\left({l}_{m},T\right)\parallel\:f\left({l}_{p},T\right)\right)$$

where $\:{D}_{ML}$ is ML divergence between two distribution probabilities $\:f\left({l}_{m},T\right)\:\text{a}\text{n}\text{d}\:f\left({l}_{p},T\right)$. Using additional supervision and regularization via soft labels with higher entropy. The loss function $\:{L}_{i\:}$for each client *i* combines its own empirical with distillation loss, which is controlled by a hyperparameter. The client adjusts its local weights based on its local objective *Fi ()* in RMPFD. Training involves using SGD with a learning rate denoted as $\:\eta\:$ to identify the model parameters that maximize accuracy on the training and test datasets.

The mechanisms of RMPFD are detailed in Algorithms 1 and 2. This framework highlights the collaborative effort between the server and edge devices to develop personalized models with reduced computational overhead. The server initializes the global model and supervises its distribution and aggregation. Selected clients then independently train their models and return their aggregated parameters to the server. Subsequently, the server establishes parallel local training and supplies the clients with the revised local models. After each training round, the server aggregates all received models to generate an updated global model. Algorithm 2 outlines the personalized training process utilizing knowledge distillation within RMPFD. It illustrates how clients conduct local model training to achieve a balance between generalization and personalization. To prevent privacy leakage, adaptive gradient clipping is employed to restrict the sensitivity of model updates, thereby protecting against adversaries gleaning private information. Adaptive Gaussian noise added to the clipped gradients before updating model soft-labels, following the DP-SGD technique^52^. The overall loss function combines the supervised learning elements with distillation loss, allowing local models to generalize while enabling clients to retain unique model characteristics benefiting from federated knowledge sharing.

**Algorithm 1:**
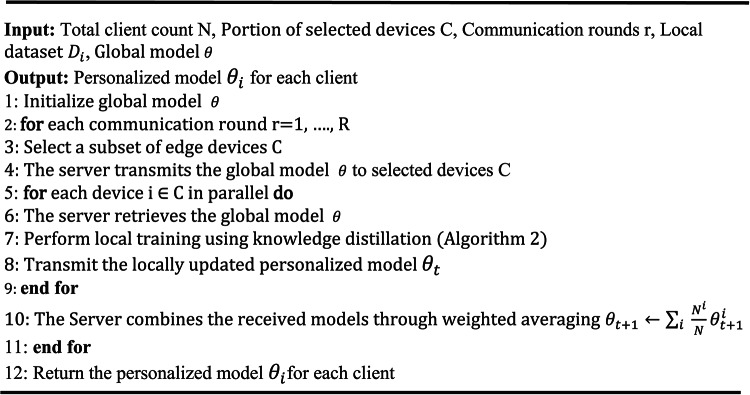
RMPFD Framework.

**Algorithm 2:**
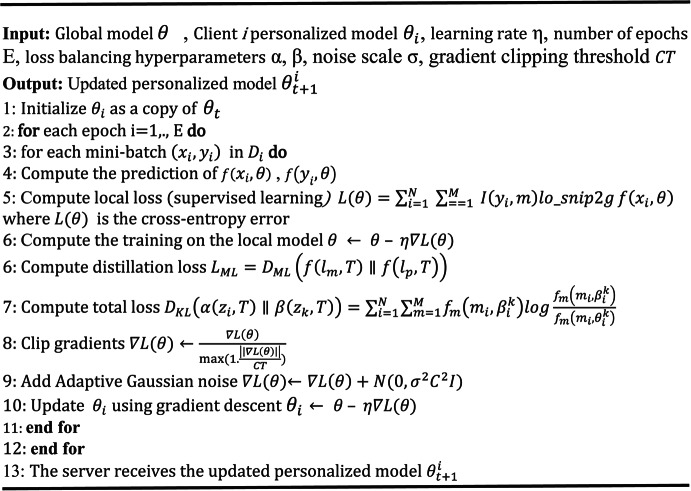
Personalized Training with Knowledge Distillation.

**Algorithm 3:**
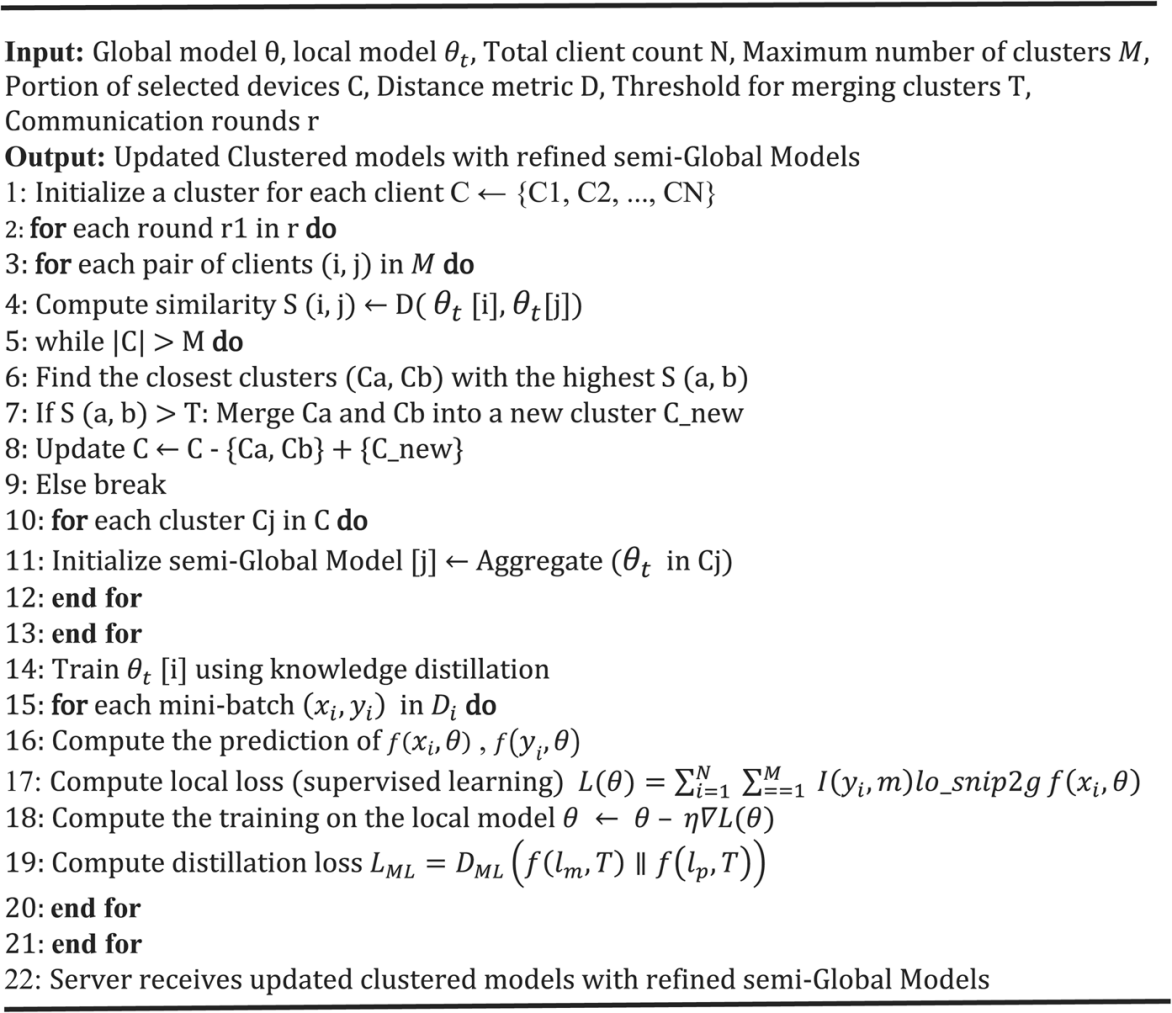
Adaptive Hierarchical Clustering in RMPFD framework

Integrating the hierarchical clustering and knowledge distillation approach for refining semi-global models ensures efficient aggregation, improves model adaptation to heterogeneous data, and enhances personalization in FL. Algorithm 3 shows a detailed pseudo-code representation of the hierarchical clustering mechanism in the RMPFD framework. This algorithm enhances PFL by dynamically grouping clients based on the similarities of their models and enables the development of semi-global models. Clients are iteratively clustered based on these similarities until a specified number of clusters (*M*) is achieved or a similarity threshold (*T*) is met. Once the clusters are formed, knowledge distillation is utilized. Each cluster employs a semi-global model by aggregating its local models, which act as teacher models. Clients refine their local models using these semi-global models, optimizing them through divergence loss $\:{L}_{ML}$. In the final stage of personalization and training, clients leverage semi-global knowledge, ensuring effective personalization while improving generalization across diverse data distributions. This adaptive clustering strategy significantly boosts model efficiency, robustness, and convergence speed in FL environments. In the following section, the factors contributing to the effectiveness of RMPFD will be examined.

### Data heterogeneity

In traditional FL, all clients must share a single global model but cannot modify their local models. However, in some cases, clients may need to develop a tailored local model to suit unique application circumstances. FL performance in limited data scenarios can be challenging. To address the challenge of insufficient raw training data, collaboration with other clients is essential to aggregate knowledge to improve the generalization of the local model. Furthermore, by drawing inspiration from deep mutual learning^53–55^, principles, and employing different network architectures for local and global models, both sides can leverage each other’s learning experiences. As a result, the global model can enhance its performance by learning from the local model.

In this study, the RMPFD is introduced as an innovative adaptive hierarchical CFL approach for addressing privacy and model heterogeneity by grouping clients based on the similarity of their local models. The RMPFD aims to train CFL models using knowledge distillation, and federated meta-learning techniques, enabling personalized historical knowledge sharing. This process ensures semi-global model creation that more accurately reflects the data characteristics of each cluster, rather than depending on a single global model. The hierarchical clustering mechanism significantly enhances personalization by allowing clients within the same group to benefit from aggregated knowledge that closely aligns with their local data distributions. These semi-global models act as teacher models during the knowledge distillation process, enabling clients to refine their personalized models without sharing data, thus ensuring privacy. Furthermore, by clustering clients before aggregation, RMPFD minimizes redundant updates, resulting in faster convergence and improved scalability of FL for practical applications where communication and computational resources are limited.

In subsequent rounds, PM-supervised local training is implemented for all clients to preserve personalized historical knowledge. Following this training, the $\:{\theta\:}_{t+1}^{i}$ is updated with the aggregated parameters of $\:{\beta\:}_{t}^{k}$ model in the current round to retain transferred knowledge. There are several techniques to facilitate the transfer of learning from a previously trained model to a new one. These techniques include fine-tuning, progressive learning, knowledge distillation, and FedMeta. The RMPFD framework utilizes FedMeta and knowledge distillation to treat each federation as a meta-distribution. The training process is divided into two stages: knowledge distillation and personalization. Following this approach, the RMPFD is required to calculate mimicry loss and traditional supervised learning loss. The mimicry loss reflects the soft labels of the $\:{\theta\:}_{i}^{k}$, while the supervised learning loss pertains to $\:{\beta\:}_{i}^{k}$. This methodology becomes particularly effective when the temperature threshold *T* is precisely set to one.

6 $$\:f\left({z}_{i},T\right)=f\left(m,{\theta\:}_{i}^{k}\right)$$

7 $$\:f\left({z}_{k},T\right)=f\left(m,{\beta\:}_{i}^{k}\right)$$

The KL distance from $\:f\left({z}_{i},T\right)$to$\:f\left({z}_{k},T\right)$ can be expressed as follows:

8 $$\:{D}_{KL}\left(f\left({z}_{i},T\right)\parallel\:f\left({z}_{k},T\right)\right)={\sum\:}_{i=1}^{N}\:{\sum\:}_{m=1}^{M}\:{f}_{m}\left({m}_{i},{\beta\:}_{i}^{k}\right)\text{l}\text{o}\text{g}\frac{{f}_{m}\left({m}_{i},{\beta\:}_{i}^{k}\right)}{{f}_{m}\left({m}_{i},{\theta\:}_{i}^{k}\right)}$$

In the context of RMPFD, the local model $\:{{\upbeta\:}}_{t}^{i}$ depicts the calculation of the loss function and the $\:\nabla\:{\theta\:}_{t}^{i}$ is as follows:

9 $$\:\nabla\:{\theta\:}_{t}^{i}={\theta\:}_{t}^{k}-{\theta\:}_{t}=\frac{\alpha\:{L}_{{\theta\:}_{t}^{i}}\left(f\left(x,{\theta\:}_{l},{\stackrel{\prime }{f}}_{t}^{i}\right),y\right)}{\alpha\:{\theta\:}_{t}}$$

The RMPFD meta-learning framework seeks to map data distributions to meta-distribution models through a set of learnable parameters optimized alongside the model parameters. A primary basis in meta-learning is that this meta-distribution can effectively reflect the function and characteristics of the model. As a result, leveraging learnable parameters to construct losses can enhance the efficiency of knowledge distillation^29^. Research on mutual learning^18^ confirms it can facilitate knowledge transfer between models and offer dynamic regularization. Overall, the RMPFD enhances the diversity and fluency of the learned labels, captures semantic relationships between classes, and thus improves personalized local models for the downstream task of knowledge transfer.

### Differential privacy

FL facilitates sharing model parameters and soft labels, enabling knowledge transfer without revealing sensitive training data. However, the latest research has indicated that sharing soft labels can expose certain information about the training data, resulting in potential privacy breaches. Malicious servers could exploit this vulnerability through gradient leakage attacks, allowing them to reconstruct sensitive information. An adversary reconstructs private training data in these attacks by analyzing the gradients transmitted during model updates. Differential Privacy (DP) mechanisms are integrated into FL frameworks to mitigate these risks, ensuring data privacy while preserving model utility.

#### Gradient leakage attack mechanism

During this process, the server transmits the global model $\:{\theta\:}_{t}$ to the edge devices to train it on private datasets *(x*,* y).* The edge devices return the updated model parameters $\:{\theta\:}_{t}^{i}$, which can calculate the gradients both before and after training. This enables the server to update the global model by inferring the characteristics of the training data. The gradient update is computed as follows:

10 $$\:\nabla\:{\theta\:}_{t}^{i}={\theta\:}_{t}^{i}-{\theta\:}_{t}=\frac{\alpha\:{L}_{{\theta\:}_{t}^{i}}\left(f\left(x,{\theta\:}_{t}\right),y\right)}{\alpha\:{\theta\:}_{t}}$$

The malicious server initializes the input $\:{x}^{\text{*}}$ and labels $\:{y}^{\text{*}}$at random to start the assault. After that, it trains using this “dummy data” ($\:{x}^{\text{*}}{,\:y}^{\text{*}}$). Following the training, to acquire further information about the training data, the server computes the “dummy gradients” and gets $\:{\theta\:}_{t}^{\text{*}}$. By analyzing the changes in the model parameters, the adversary reconstructs sensitive data:

11 $$\:\nabla\:{\theta\:}_{t}^{\text{*}}={\theta\:}_{t}^{\text{*}}-{\theta\:}_{t}=\frac{\alpha\:{L}_{{\theta\:}_{i}^{\text{*}}}\left(f\left({x}^{\text{*}},{\theta\:}_{t}\right),{y}^{\text{*}}\right)}{\alpha\:{\theta\:}_{t}}$$

Comparing $\:\nabla\:{\theta\:}_{t}^{\text{*}}$ with $\:\nabla\:{\theta\:}_{t}^{i}\:$reveals key information about the original training data, potentially allowing the attacker to recover personal details, such as medical records or financial transactions. The backdoor attack model involves malicious users inserting hidden backdoors into a targeted model through poisoned updates^56^. This leads the model to misbehave with certain inputs while still performing normally in other situations.

#### FL for gradient protection

FL proposes controlled arbitrariness in the learning process to prevent gradient leakage, ensuring that individual data points do not significantly impact the model updates. DP enhances data security in FL by preventing gradient leakage attacks while maintaining model accuracy. By combining noise injections, gradient clipping, and secure aggregation, FL can be effectively deployed in privacy-sensitive applications.

One common DP technique of FL is the adaptive (DPFL-AGN) scheme^57^. It specifically added Gaussian noise in the training process to protect the client’s raw data. Adding gaussian noise Ɲ(0, *σ*^2^) with mean 0 and variance $\:{\sigma\:}^{2}$ to gradient updates before transmission as follows:12

CFL has emerged as an effective framework for facilitating various intelligent IoT applications. However, the model gradients or weights may still contain private information, which poses a risk of inference attacks^58^. To prevent individual data points from disproportionately influencing model updates, a local DP model is employed, incorporating a gradient clipping threshold $\:CT$. This mechanism restricts the impact of any single client’s data on the overall model. Consequently, it successfully strikes a balance between privacy and utility, achieving superior performance:

13 $$\:\nabla\:L\left(\theta\:\right)\leftarrow\:\frac{\nabla\:L\left(\theta\:\right)}{{max}(1.\frac{\left|\left|\nabla\:L\left(\theta\:\right)\right|\right|}{CT})}$$

The rise of the IoT has significantly enhanced the quality of services in various applications including smart cities, healthcare, and edge computing. However, the heterogeneity of interconnected IoT devices within smart cities reinforces the risk of malicious attacks, necessitating secure and privacy-preserving solutions. One effective strategy to tackle these challenges is Homomorphic Encryption, a cryptographic technique that allows computations on encrypted data without n eed for decryption^59^. By utilizing this method, clients can conduct local aggregation and transmit encrypted updates instead of sending raw data, thereby improving security and privacy:

14 $$\:{\theta\:}_{t}=\frac{1}{N}\:{\sum\:}_{i=1}^{N}Encrypt\:(\nabla\:{\theta\:}_{t}^{i})$$

In RMPFD, the local model utilizes a differential privacy perturbation mechanism^60^ adding adaptive noise to the shared parameters makes it more difficult for adversaries to reconstruct clients’ private data, thereby enhancing data privacy during training.

#### Computational trade-offs in DP for RMPFD

While DP provides robust privacy guarantees in FL, its integration introduces computational overhead that must be carefully managed. The RMPFD framework balances privacy, computational efficiency, and model performance, particularly in real-world FL scenarios like healthcare, smart cities, and IoT applications. Adding Gaussian noise to model updates and implementing gradient clipping introduces extra computational steps, increasing training time and resource consumption. Computational efficiency is a significant concern in constrained environments like edge devices in IoT or medical imaging systems in healthcare. RMPFD mitigates this overhead by:

i. *Adaptive noise injection*: Instead of applying a fixed noise level, RMPFD dynamically adjusts the privacy budget based on model sensitivity to reduce unnecessary perturbations while preserving privacy.
ii. *Optimized gradient clipping*: RMPFD ensures that gradients are not excessively distorted by selecting an appropriate clipping threshold $\:CT$, maintaining training stability while preventing excessive computational costs.

DP mechanisms inherently introduce a trade-off between privacy and model performance. Excessive noise addition can degrade model accuracy. To address this, RMPFD incorporates:

i. *Knowledge distillation*: RMPFD leverages soft label distillation instead of relying solely on raw model updates, allowing personalized models to learn useful representations without direct exposure to raw gradients.
ii. *Meta-learning strategies*: RMPFD enhances generalization by treating each client as a meta-distribution, enabling effective adaptation in non-IID scenarios.

#### Real-world applications: balancing privacy and efficiency

In healthcare sector, the privacy-sensitive domains like medical imaging or electronic health records (EHRs**)** ensures that patient data remains secure during FL training using DP. However, to maintain diagnostic accuracy, RMPFD fine-tunes the trade-off by using selective noise application to high-impact model parameters rather than uniform noise addition.

**Fig. 3:**
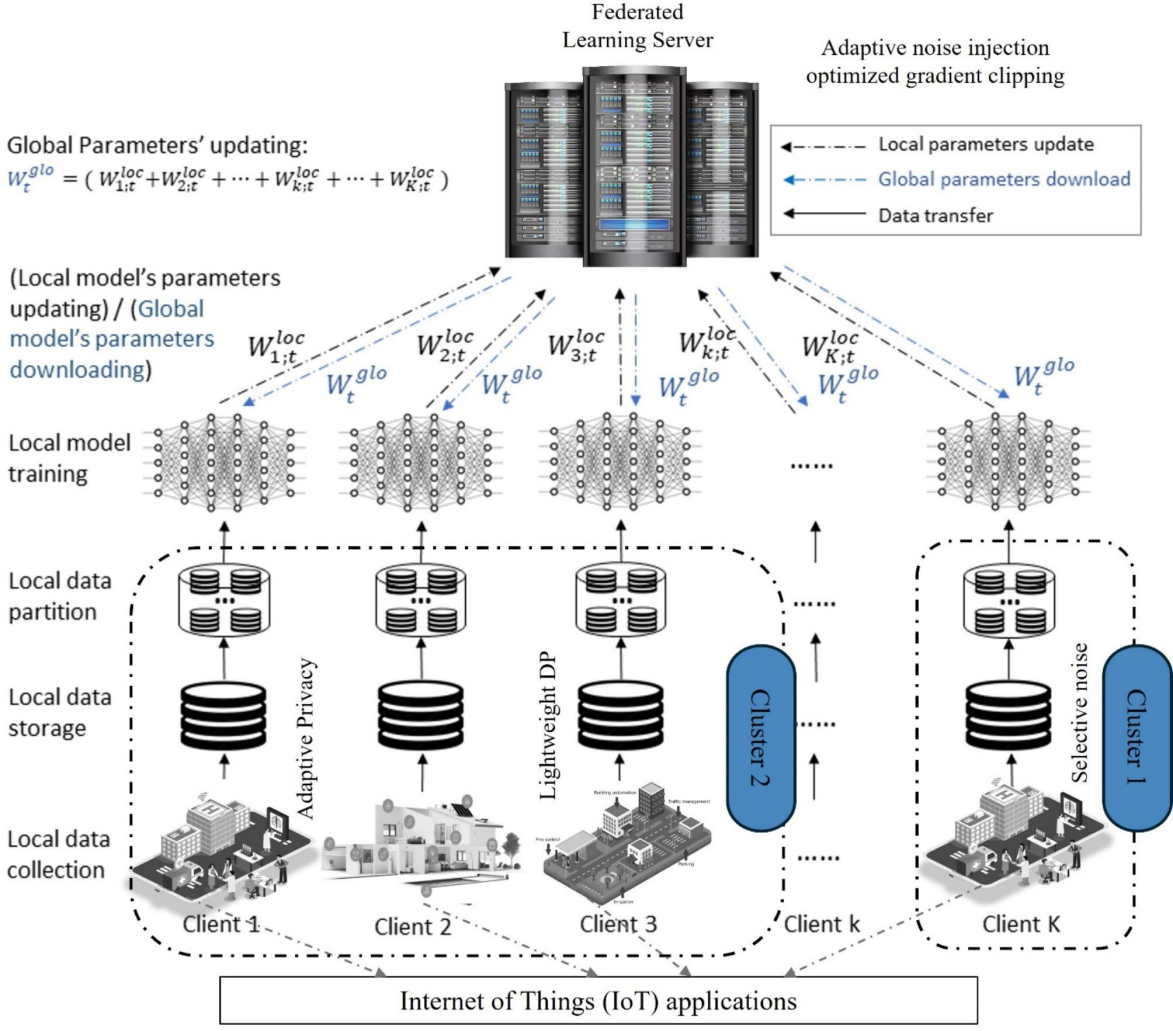
The conceptual diagram of the RMPFD System on different real-world applications.

For edge computing applications, RMPFD ensures efficiency by offloading encryption and DP operations to IoT devices or federated nodes with higher capabilities that reduce latency and energy consumption. RMPFD focuses on developing adaptive privacy mechanisms based on real-time sensitivity analysis by leveraging pre-trained models using federated transfer learning and meta-learning techniques to reduce the need for extensive local training, thus minimizing DP-induced performance degradation. RMPDF focuses on exploring lightweight encryption methods to reduce secure aggregation overhead. By strategically optimizing privacy mechanisms and computational efficiency, RMPFD ensures scalable and secure FL deployment across diverse real-world applications. Figure 3 presents an overview of the RMPFD system and its DP mitigation techniques in different real-world domains.

### System design architecture

**Fig. 4:**
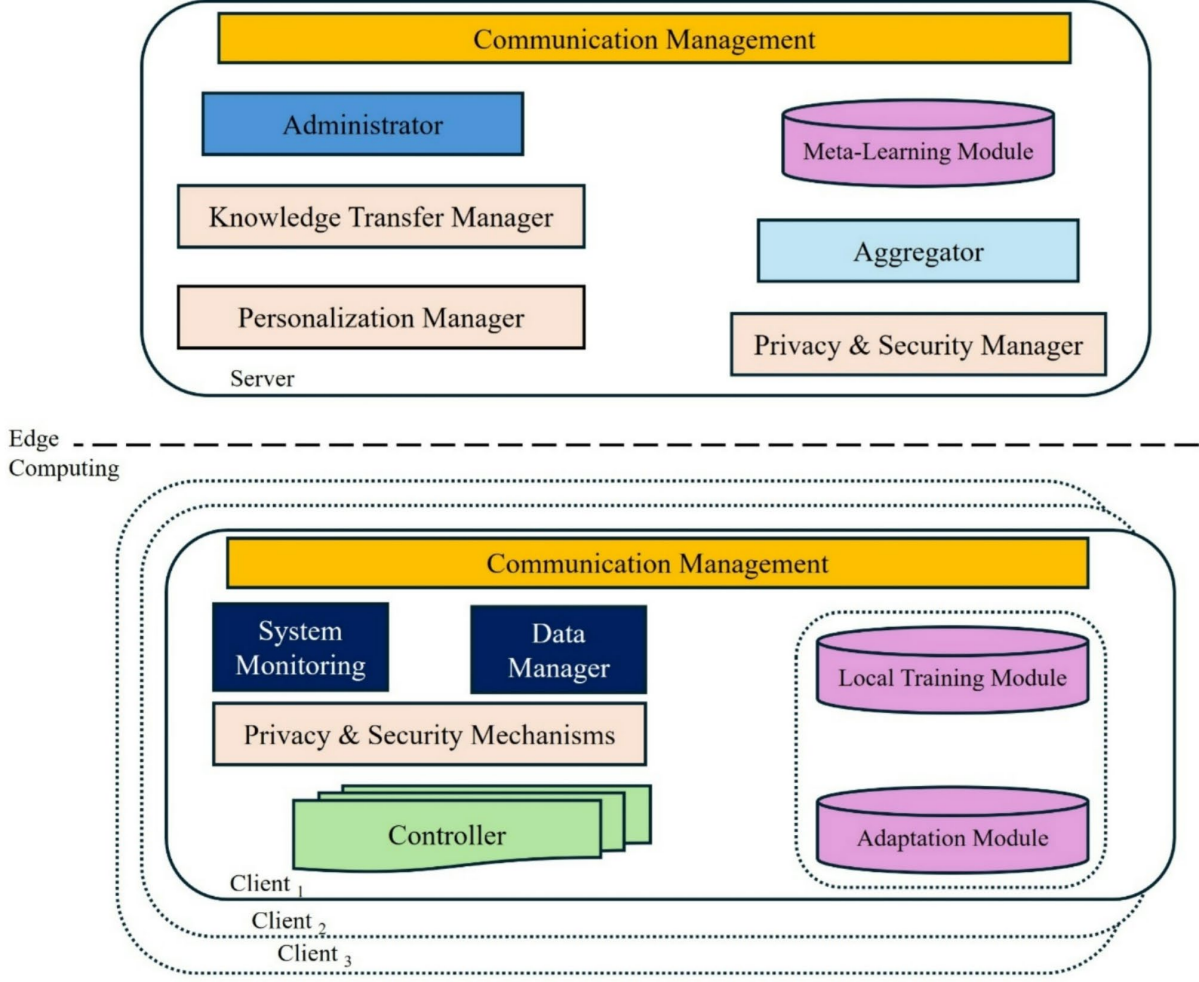
High-level Illustration of the System architecture for the RMPFD workflow with Cloud Server and Edge Devices.

The RMPFD model architecture enables effective training and personalization through collaborative work between the cloud server and edge devices. The cloud server manages the learning process by aggregating model updates and delivering the global model. Meanwhile, edge devices train local data and develop adaptive local models. The RMPFD system features a modular structure that separates training, communication, and storage components. This architecture provides flexibility in managing diverse data types, supporting the integration of pre-trained models, and employing advanced algorithms. A high-level overview of the system design and architecture is presented in Fig. 4. On the cloud server side, the components include:

i. *Administrator*: This component is responsible for maintaining and managing the global model, updating and distributing it to all edge devices using meta-learning techniques, to enable rapid adaptation for each client.
ii. *Aggregator*: The secure aggregator combines models received from each client to update the global model. It is activated once all participating clients have submitted their models or when a predetermined time constraint is reached. It encrypts updates before aggregation to ensure the privacy of client data.
iii. *The communication manager*: The communication manager handles communication rounds, interruptions, and model distribution. Additionally, this component tracks device availability and network conditions.
iv. *Clustering and personalization manager*: This component classifies clients into clusters based on similarity or task relevance. Clustering allows the use of multiple versions of the global model tailored to specific user groups.
v. *Meta-learning module*: This module employs meta-learning techniques such as Model-Agnostic Meta-Learning (MAML) or Reptile^61^ to pre-train the global model.
vi. *Knowledge transfer manager*: This component directs the knowledge transfer between global and personalized models through the Knowledge Distillation techniques.
vii. *Privacy and security manager*: This component guarantees privacy when clients transmit gradients or soft labels to the server by utilizing a differential privacy perturbation mechanism^60^ to prevent gradient leakage attacks.

On the edge device side, the module consists of the following components:

i. *Controller*: This essential component supervises local processes and issues directives to other modules on the edge device.
ii. *System monitor*: The system monitor collects extensive statistics about the device, including metrics on battery performance and system load. The controller then relays this information to evaluate the feasibility of accepting work requests from the server.
iii. *Data manager*: This module manages the personalized model on the edge device. During each FL round, it receives the global model, conducts local training, and sends the model updates back to the cloud server.
iv. *Local training module*: Performs training on local data. This module also implements personalization layers and regularization techniques and prevents overfitting on limited local data.
v. *Communication manager*: Handles communication with the cloud server. It manages efficient and secure transmission of updates, often minimizing data transfer to conserve bandwidth and power by compressing model updates to reduce transmission costs.
vi. *Adaptation module*: It helps the local model rapidly adapt to the user’s data with minimal updates while using meta-learning techniques like MAML to initialize the model so that only a few gradient steps are needed for effective personalization.
vii. *Privacy and security mechanisms*: These protect data privacy on the edge device. Common methods are used including differential privacy and Secure Aggregation Protocols.

In the context of the RMPFD approach, there is minimal system overhead compared to the conventional FL framework, and it offers two methods to obtain a predictive model $\:{\theta\:}_{t}$ on client data. The first approach involves storing past predictions of $\:{\theta\:}_{t}$, thus, reducing computational load by reusing these predictions in subsequent rounds requires additional storage capacity on the client device. Alternatively, clients may retain the past model locally providing greater flexibility, which may demand more GPU memory. This approach is well-suited to maintain and deliver global and tailored models to clients during the model download phase in high-availability environments. Clients thus benefit from leveraging the global model for generalized updates, while using their personalized model for individualized refinement, facilitating efficient and adaptive personalization. Consequently, the RMPFD architecture effectively balances resource constraints with the need for tailored model performance across diverse client devices.

### RMPFD analysis

The RMPFD divides the training and test datasets into smaller each associated with a specific client and organizes them into Data_Loaders according to the designated number of clients. Following dataset partitioning, the model is defined with key functions for training and evaluation, including set_parameters and get_parameters. To implement the Fed_Client class, a subclass of flwr_Client is created. Specifically, a NumPyClient is used, implementing essential methods such as get_parameters, and evaluating these methods to log additional client-specific information through a client ID (CID). For server-side initialization, RMPFD begins by obtaining initial parameters, either by requesting them from a random client or by setting parameters directly through the FedAvg strategy to avoid client dependency. The start simulation function facilitates flexible control over the training environment, allowing specification of the client function (client_fn), number of clients to simulate, training rounds, and CFL strategy. The strategy here captures the specific CFL algorithm employed, such as FedAvg, with modifications tailored to support personalized learning in RMPFD.

The RMPFD framework supports centralized (server-side) and federated (client-side) evaluation approaches. Server-side evaluation is straightforward and does not necessitate transferring models back to clients, while client-side evaluation, though more complex, allows for broader data coverage, increasing robustness. Configuration settings can be transmitted from the server to clients through a dictionary, which can be used to fine-tune parameters such as training epochs and evaluation metrics. The RMPFD’s flexible strategy structure permits passing arbitrary values between the server and clients. Clients can return custom metrics or values through a dictionary in the fit and evaluate methods, enabling richer monitoring and adaptation of the CFL process.

Assuming that data is divided into 1000 segments, each containing 45 training instances and 5 validation instances. Clients are set up to perform extended local training by executing 5 local epochs. Moreover, the number of devices participating in each training round was adjusted by setting the fraction fit to 0.05 indicating that only 5% or 50 clients are active per round. This allows RMPFD to strike a balance between computational efficiency and model diversity. Collaboration between the Flower Virtual Client Engine^62^ and the RMPFD enables extensive simulations for client pools. It provides valuable insights into how different initialization, evaluation, and configuration settings affect performance in high-dimensional environments. As a result, RMPFD supports comprehensive and scalable transfer learning that effectively adapts to resource constraints and the diverse needs of clients.

## Experimental work and discussion

This section provides a detailed analysis and performance evaluation of the RMPFD framework through an extensive experimental setup. Two distinct FL scenarios were designed to evaluate RMPFD’s adaptability and resource efficiency across various client configurations. In the first scenario, 20 clients participated fully, achieving a 100% participation rate in over 50 communication rounds. In the second scenario, 100 clients engaged with a participation rate of 10% spanning 100 communication rounds. The selected clients conducted 5 local training epochs in each round, emphasizing RMPFD’s iterative and personalized training methodology. To improve robustness, the average test accuracy across clients was calculated by averaging the results over three random seeds. By reporting both means and standard deviations, this analysis provides a thorough understanding of RMPFD’s effectiveness in balancing personalized adaptation with computational resource management under diverse scenarios of client participation and training rounds. Consequently, it highlights the RMPFD’s suitability for high-dimensional federated environments.

### Datasets and setting

Three standard datasets are used to evaluate the efficiency of the RMPFD framework, CIFAR- 10, CIFAR- 100^63^, Fashion-MNIST (FMNIST)^64^, and Enron email^65^ datasets. Both CIFAR- 10 and CIFAR- 100 datasets are commonly utilized for image classification tasks. FMNIST is a subset of the MNIST dataset, serving as a shared private dataset in this analysis. The Enron Email Dataset is a non-IID dataset that comprises real-world email communications from the Enron corporation. The experiments are conducted on IID and non-IID data configurations for each dataset. In the IID setup, each client receives data with an evenly balanced distribution across classes, ensuring consistent class representation among clients. In contrast, the non-IID configuration reflects a more realistic, heterogeneous data distribution where each client trains samples from a single source, while in testing, clients must classify samples originating from multiple sources. This arrangement models common non-IID conditions in federated settings where individual data distributions vary significantly. The Flower framework provides baselines utilizing different data-splitting strategies, encompassing non-IID strategies based on label, feature, and quantity skew^66^. Further details on the dataset characteristics are provided in Table 2.

**Table 2:** Training and testing dataset characteristics.

Public dataset	No. of samples	No. of classes	Private	Collaborative task
MNIST	70,000	10	Letters from certain classes (Fashion-MNIST)	IID: Image Classification
			Letters from one writer (Fashion-MNIST)	Non-IID: Image Classification
CIFAR- 10	60,000	10	Subclasses (CIFAR100)	IID: Image Classification
			Super classes [0–5] (CIFAR100)	Non-IID: Image Classification
Enron email	40,000	2	Emails from different users	Non-IID: Text Classification

In this experimental setup, the CIFAR- 10 dataset is employed as a public resource accessible to all clients, whereas the CIFAR- 100 dataset acts as a private resource. CIFAR- 100 comprises 100 subclasses, organized into 20 superclasses that are group-related categories. For example, the"carnivore” superclass includes related subclasses such as lion, tiger, leopard, wolf…. etc. In the case of IID distribution, each client receives data consisting of a variation of subclasses, ensuring a balanced representation of training. In the non-IID distribution, training data is confined to a single subclass within a specific superclass. During the testing phase, clients are tasked with classifying images from the wider superclass, despite having been trained in only one subclass. For instance, a client trained solely in “bear” images must accurately identify other animals in the “large carnivore” superclass, such as tigers, when they encounter them during testing. This design strictly resembles real-world FL scenarios, where client data distributions can vary significantly, involving the training and evaluation phases.

### Model structure and pre-train strategy

**Fig. 5:**
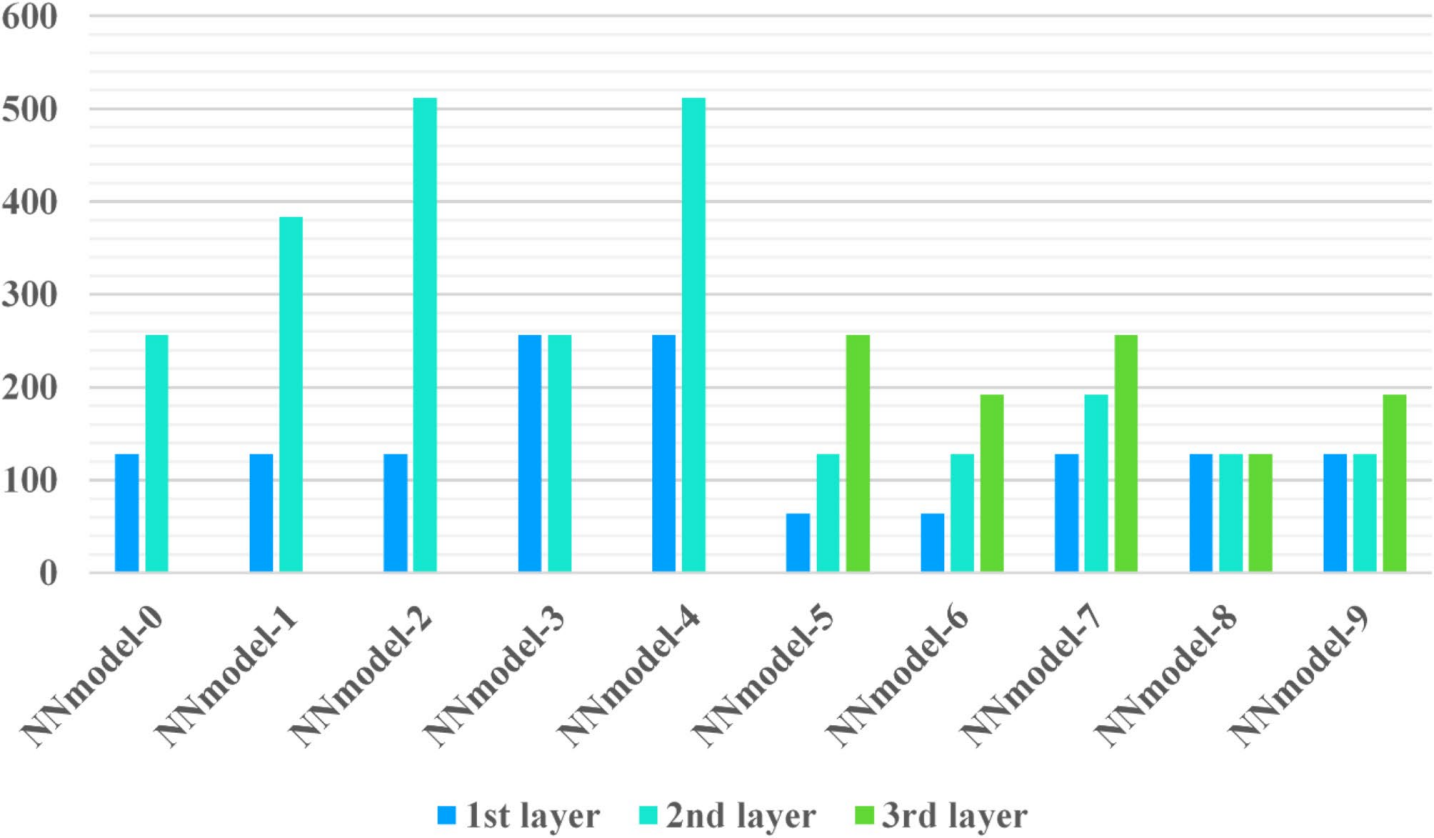
NN models architecture performance for the F-MNIST dataset.

**Fig. 6:**
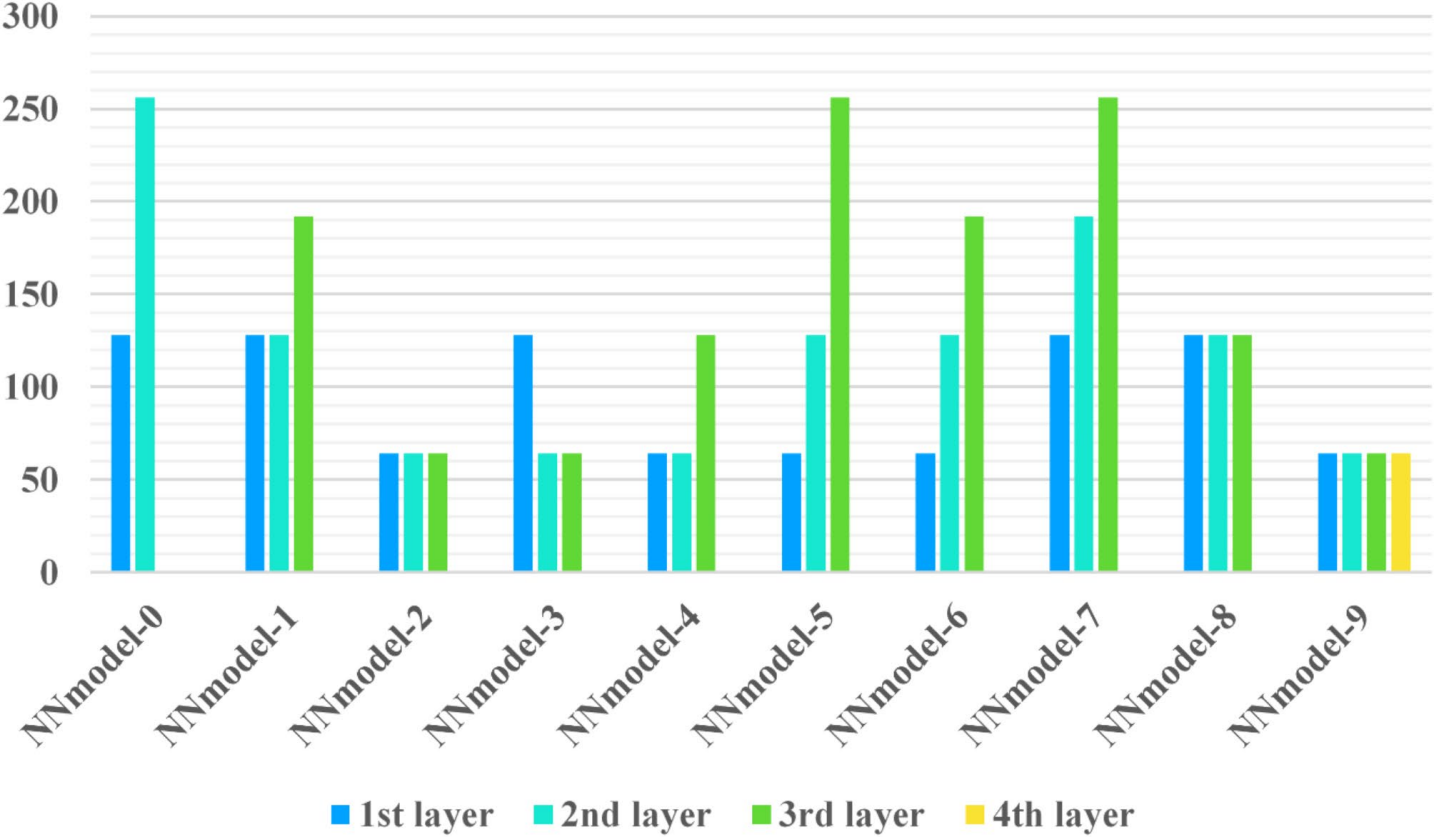
NN models architecture performance for CIFAR10/CIFAR100 dataset.

In this experimental framework, a simple convolutional neural network (CNN) is employed for the FMNIST dataset and 5-layer CNNs for the CIFAR dataset, on the other hand, a more complex 5-layer CNN architecture is utilized for both CIFAR- 10 and CIFAR- 100 datasets. To reflect model diversity, configuring distinct model structures across 10 clients, where each client’s model varies based on the number of convolutional layers and channels. The performance of these architectures on the FMNIST, CIFAR- 10, and CIFAR- 100 datasets is illustrated in Figs. 4 and 5. The training process begins with all clients initializing their respective models and engaging in a pre-training phase on the public dataset until convergence. Following this phase, each client advances to fine-tune its model on its private dataset for several additional iterations. During pre-training, models generally achieve high accuracy, ranging from 98 to 99% on the MNIST dataset and between 71.6 and 78.2% on the CIFAR datasets, as summarized in Tables 3 and 4. This phased training approach—combining pre-training on public data with private fine-tuning—enables clients to establish a robust baseline before adapting to their unique data distributions. Figures 5 and 6 show the performance of neural network models architecture for the F-MNIST, and CIFAR10/CIFAR100 datasets.

**Table 3:** Accuracy ranges for the FMNIST dataset.

Name of model	Dropout	Pre-train accuracy (%)
NNmodel- 0	0.2	98.6
NNmodel- 1	0.2	98.3
NNmodel- 2	0.2	98.8
NNmodel- 3	0.3	98.4
NNmodel- 4	0.4	98.5
NNmodel- 5	0.2	99.0
NNmodel- 6	0.2	98.9
NNmodel- 7	0.2	99.1
NNmodel- 8	0.3	99.0
NNmodel- 9	0.3	99.0

**Table 4:** Accuracy ranges for CIFAR10/CIFAR100 datasets.

Name of Model	Dropout	Pre-train accuracy
NNmodel- 0	0.2	71.7% ± 1.8
NNmodel- 1	0.2	72.4% ± 1.4
NNmodel- 2	0.2	74.3% ± 0.7
NNmodel- 3	0.3	72.1% ± 2.3
NNmodel- 4	0.4	71.6% ± 2.9
NNmodel- 5	0.2	77.3% ± 0.6
NNmodel- 6	0.2	76.7% ± 1.2
NNmodel- 7	0.2	75.0% ± 2.0
NNmodel- 8	0.3	78.2% ± 0.4
NNmodel- 9	0.2	77.3% ± 0.7

#### Baselines approaches

To assess the performance of RMPFD, it benchmarked against several established FL methods, which are divided into two main classes:

##### Non-personalized FL approaches

i. FedAvg^67^: This method learns a global model by averaging local model parameters across all clients.
ii. FedProx^11^: An enhanced version of FedAvg incorporates a proximal term designed to restrict local updates, ensuring they remain closely aligned with the global model. This modification is especially beneficial for handling non-IID data distributions effectively.
iii. CMFL^24^: This approach improves communication efficiency and speeds up the convergence of the FL process by minimizing unnecessary data updates.
iv. FedDrop^25^: An enhanced version of traditional dropout technique used for model pruning. It helps mitigate overfitting by reducing communication overhead and the computational load on devices.
v. Weighted FedCOM^26^: It is an advanced approach that combines weighted aggregation with model compression, significantly reducing communication overhead.

##### Personalized FL approaches

i. FedPer^34^: This method partitions the neural network into a shared “body” and a client-specific “head.” The body is aggregated across clients to promote global representation, while the head is tailored to the individual needs of each client.
ii. LG-FedAvg Implements a parameter-decoupling approach, where clients independently learn compact local representations while sharing a global head across the federation.
iii. pFedMe^68^: Uses Moreau envelopes as a regularized loss function, facilitating the maintenance of a balance between the personalized and global models for each client.
iv. FedFomo^33^: Determines an optimal, weighted combination of models for each client using first-order approximations. To evaluate compatibility with local objectives, FedFomo requires clients to maintain an additional validation set for assessing other clients’ models.

#### Hyperparameter configuration

For all methodologies employed, the learning rate was established at 0.001. The Adam optimizer was utilized, incorporating a weight decay of 1e- 5 and a momentum of 0.9. The training process was carried out with a consistent batch size of 64 across all experimental trials.

#### Implementation details

All the mentioned baselines have been implemented in PyTorch and the Flower framework^62^. PyTorch is used for the model training pipeline and data loading. Subsequently, PyTorch federated using the Flower FL framework. The server and clients were simulated in a multi-processing environment with MPI as the communication backend. All experiments were executed on a DL server distributed with four V100 GPUs, enabling efficient large-scale evaluation of RMPFD’s performance and adaptability across various FL methods in personalized and non-personalized contexts.

### Performance analysis

#### Comparative accuracy analysis

The proposed RMPFD framework was evaluated against baseline methods using the MNIST and CIFAR benchmark datasets. Tables 5 and 6 present the accuracy of client models during the collaborative learning process with both RMPFD and the baseline methods. Table 5 illustrates the consistent superiority of RMPFD over other approaches across various datasets and client configurations. Specifically, RMPFD achieved average accuracies of 99.2% and 99.0% on the Fashion-MNIST dataset, outperforming the baseline method, which recorded accuracy of only 90.2% and 86.8%, respectively. These experimental results demonstrate that RMPFD surpasses the baseline methods by a significant margin of over 5.00% on both datasets.

It’s worth noting that FedProx underperforms compared to FedAvg in scenarios where only some clients participate, as it tends to pull the local optimization direction towards global distribution. Additionally, FedFomo and FedPer exhibited suboptimal performance in common non-IID settings. FedFomo’s strict aggregation strategy limits its ability to leverage diverse data distributions from other clients, while FedPer’s dependency on a shared representation negatively impacts its performance. Moreover, pFedSD also demonstrated suboptimal results compared to RMPFD, reinforcing the challenges associated with reliance on shared representations. Importantly, RMPFD exhibited high performance on more complex tasks, such as those presented by the CIFAR- 100 dataset, highlighting its scalability and effectiveness for large-scale FL systems.

**Table 5:** Performance overview of models on CIFAR10/100 IID and non-IID dataset.

Datasets	No. of Clients	FedAvg	FedPer	LG-FedAvg	pFedMe	FedFomo	pFedSD	RMPFD
FMNIST	20 clients	90.2 ± 0.1	96.3 ± 0.1	95.2 ± 0.2	93.2 ± 0.1	95.4 ± 0.1	96.6 ± 0.1	99.2 ± 0.1
	100 clients	86.8 ± 0.4	95 ± 0.2	91.4 ± 0.1	92.1 ± 0.1	92.1 ± 0.1	96.0 ± 0.1	99.0 ± 0.1
CIFAR- 10	20 clients	50.4 ± 0.7	80.7 ± 0.5	78.6 ± 0.3	79.5 ± 0.3	79.3 ± 0.3	82.1 ± 0.5	90.5 ± 0.2
	100 clients	49.0 ± 0.8	78.9 ± 0.9	71.6 ± 1.5	74.6 ± 0.7	73.2 ± 0.7	80.2 ± 0.2	90.1 ± 0.3
CIFAR- 100	20 clients	32.2 ± 0.3	52.1 ± 0.2	40.7 ± 0.1	38.4 ± 0.7	44.7 ± 0.4	55.3 ± 0.3	59.2 ± 0.2
	100 clients	29.1 ± 0.2	44.5 ± 0.6	21.6 ± 0.2	27.1 ± 0.3	26.5 ± 0.3	48.9 ± 0.8	50.9 ± 0.3

**Table 6:** Performance overview of models on MNIST/FMNIST IID and non-IID dataset.

Datasets	No. of Clients	FedAvg	FedPer	LG-FedAvg	pFedMe	FedFomo	pFedSD	RMPFD
FMNIST	20 clients	75.7 ± 0.3	99.4 ± 0.1	99.2 ± 0.1	98.8 ± 0.1	99.3 ± 0.1	99.4 ± 0.1	99.7 ± 0.1
	100 clients	84.1 ± 0.4	97.3 ± 0.1	95.7 ± 0.1	94.9 ± 0.1	96.1 ± 0.4	97.4 ± 0.1	98.1 ± 0.1
CIFAR- 10	20 clients	45.4 ± 0.5	91.7 ± 0.1	92.2 ± 0.4	89.7 ± 0.4	91.7 ± 0.2	92.5 ± 0.2	94.3 ± 0.2
	100 clients	43.5 ± 1.7	86.7 ± 0.2	79.3 ± 1.6	80.5 ± 1.1	81.6 ± 0.5	86.8 ± 0.3	90.8 ± 0.5
CIFAR- 100	20 clients	31.9 ± 0.2	55.6 ± 0.3	45.5 ± 0.6	38.7 ± 1.2	48.8 ± 0.1	59.5 ± 0.1	63.5 ± 0.2
	100 clients	27.9 ± 0.4	47.0 ± 0.5	18.9 ± 0.3	26.9 ± 0.3	25.2 ± 0.6	50.9 ± 0.4	60.5 ± 0.3

#### Communication efficiency

**Fig. 7:**
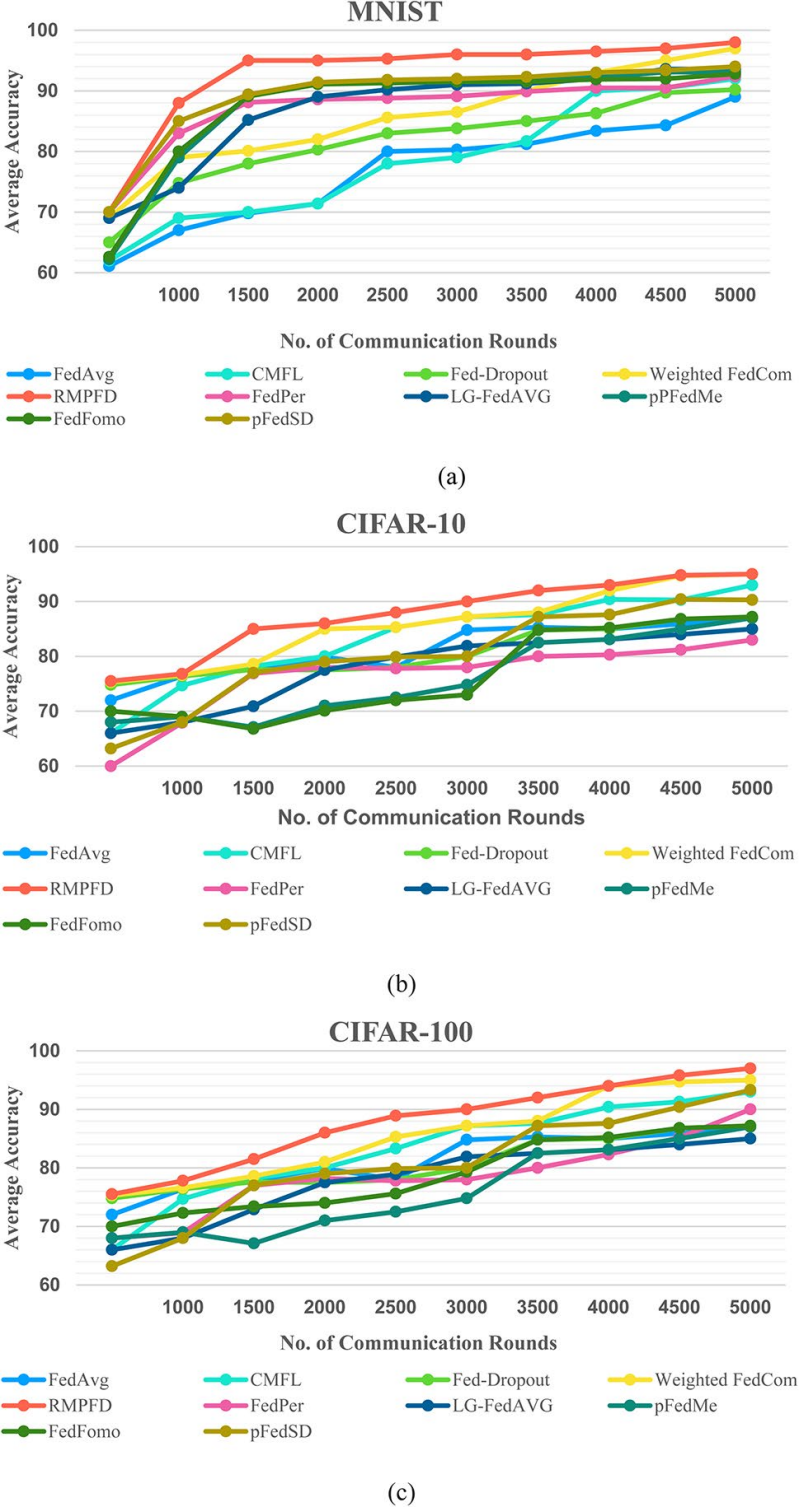
The mean generalization performance of models during the collaborative phase across the CIFAR- 10, CIFAR- 100, MNIST, and Fashion-MNIST datasets.

The personalized accuracy achieved during each training round across various datasets and configurations is depicted in Fig. 7. The learning curves illustrate that as the collaborative training phase advances, the performance of the RMPFD framework exhibits significant and consistent improvement. In contrast, the baseline methods demonstrate a temporary decline in performance following the initial rounds of communication. RMPFD ultimately surpasses the baseline methods later in the training process, benefiting from the advantages of transfer learning. On the MNIST dataset, the RMPFD framework demonstrates remarkable efficiency and effectiveness compared to various baseline methods. It achieves faster convergence and higher accuracy, surpassing FedAvg, Weighted FedCom, LG-FedAVG, and FedFomo in fewer communication rounds. By 2000 communication rounds, RMPFD reached an accuracy above 95%, while many baseline approaches are still below 90%. On the CIFAR- 10 dataset, RMPFD maintains strong performance and stability despite the more complex nature of the data. It competes closely with CMFL and pFedMe. The framework achieves steady accuracy growth, reaching around 90% by the communication round 5000, demonstrating its adaptability to high-dimensional and diverse data. In contrast, FedAvg and FedPer show slower improvement and lower final accuracy, indicating that RMPFD is more effective in ensuring high performance while preserving privacy. These results emphasize RMPFD’s capability to deliver high model accuracy with efficient communication and privacy preservation.

In a scenario with 20 clients using the CIFAR- 10 dataset, FedPer reaches its peak initially but then experiences fluctuations downward, while pFedSD maintains stability. This indicates that the instability in FedPer’s performance could be due to overfitting, which self-distillation in pFedSD helps to alleviate. Furthermore, RMPFD requires fewer communication rounds to achieve equivalent accuracy, as evidenced in Table 4, where RMPFD demonstrates convergence rates that can be up to five times faster than pFedSD in certain scenarios. This highlights RMPFD’s superior communication capabilities due to its rapid convergence. Regarding fairness among clients, the personalized performance of individual clients can vary significantly due to inherent differences in their data distributions. A detailed analysis of personalized model performance reveals that our method exhibits a lower SD across all clients in the 100-client scenario, as shown in Table 5. This indicates a more equitable distribution of performance among clients. The RMPFD demonstrates enhanced fairness, leading to improved accuracy across various clients.

**Fig. 8:**
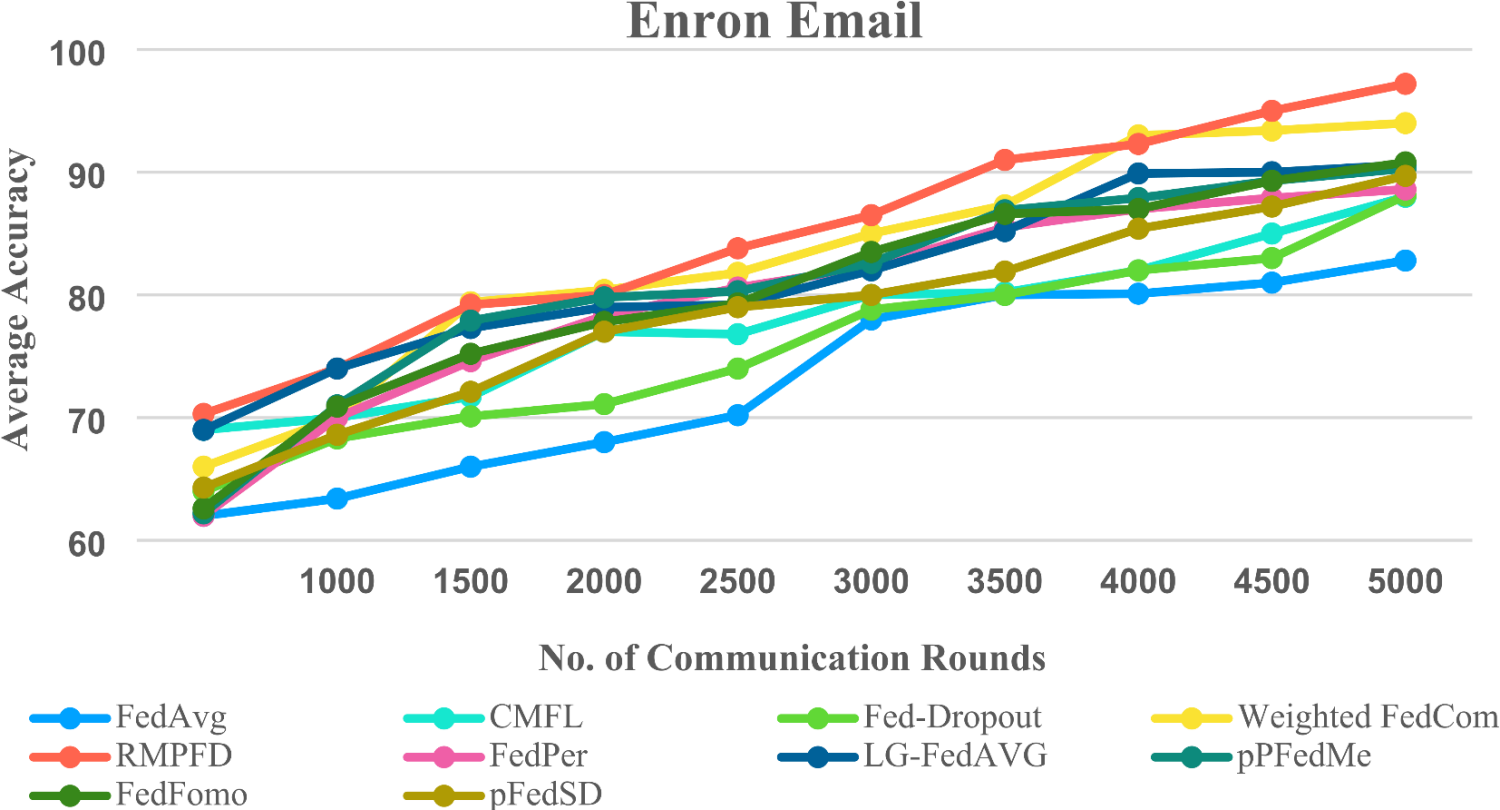
The mean generalization performance of models during the collaborative phase across the Enron Email dataset.

The average accuracy achieved during each training round on the Enron email dataset and various FL baseline configurations is depicted in Fig. 8. The RMPFD consistently outperforms the conventional FL methods across all communication rounds and exhibits superior model convergence. This advantage stems from the integration of adaptive clustering techniques with meta-learning. By round 5000, RMPFD achieves the highest accuracy, significantly exceeding that of FedAvg, CMFL, and FedPer methods. This highlights RMPFD’s effectiveness in balancing privacy, computational efficiency, and model performance. Additionally, the swift improvement in accuracy observed between rounds 1000 and 3000 highlights RMPFD’s ability to effectively utilize DP mechanisms while ensuring robust learning.

**Fig. 9:**
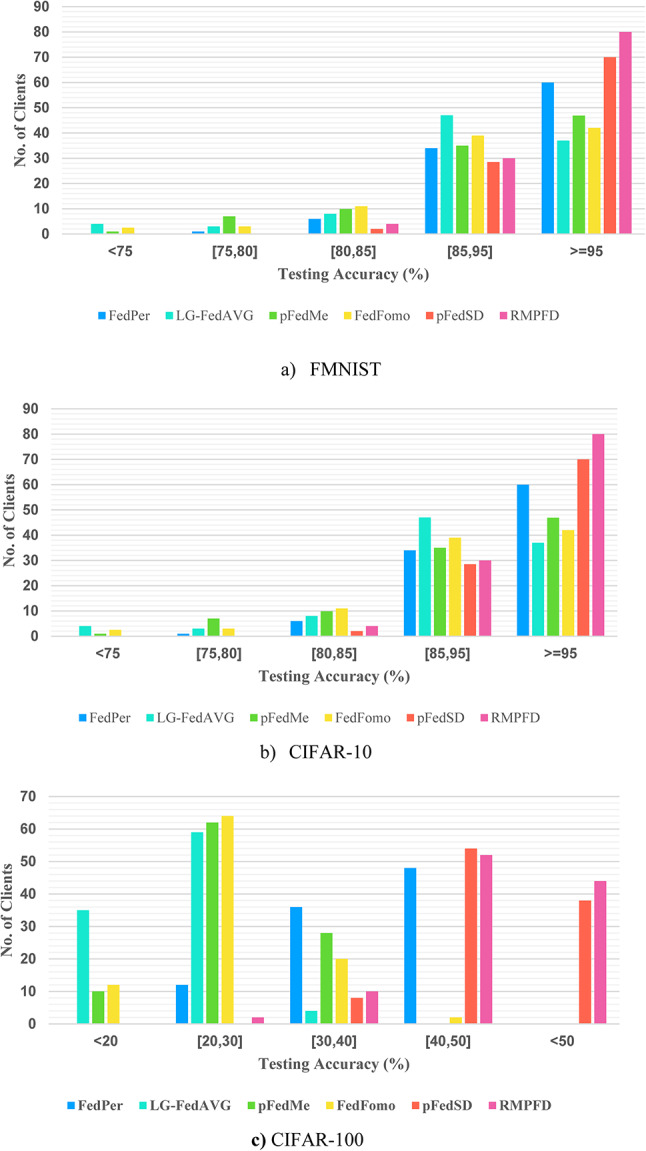
The mean experimental accuracy observed among all participating clients.

The primary objective of CFL is to minimize variations while ensuring an acceptable level of average experimental accuracy. The proposed approach demonstrates the lowest standard deviation across the FMNIST, CIFAR- 10, and CIFAR- 100 datasets. Additionally, Fig. 9 illustrates the performance distribution among all clients, revealing that RMPFD consistently achieves a higher proportion of clients with elevated testing accuracy across all datasets. Overall, RMPFD attains greater fairness and results in more clients with improved accuracy.

### Sensitivity analysis

In this analysis, the impact of varying participation ratios on the CIFAR- 100 dataset was analyzed. The findings showcased that the RMPFD consistently outperforms all baselines regardless of the participation ratio setting. Furthermore, there is an increase in the ratio from 20 to 60% resulting in enhanced accuracy for all methods due to increased training rounds for each client. However, when the ratio was elevated from 60 to 100%, some approaches, such as pFedSD, might experience an overfitting of the local model. Conversely, the performance of FedProx notably improved with the increase in participation ratio, aligned with the latest global distribution.

Moreover, the impact of heterogeneous data on the CIFAR- 100 dataset was analyzed by adjusting statistical heterogeneity levels through different concentration parameters. Across various non-IID settings characterized by high data heterogeneity, RMPFD consistently achieved superior accuracy. Even under more balanced data distributions, RMPFD maintained a clear advantage over other personalized FL approaches. These findings highlight the effectiveness and resilience of RMPFD in handling varying degrees of data heterogeneity in FL environments.

### System performance evaluation

To evaluate the efficiency of the RMPFD method, a set of performance and fairness metrics are employed. This assessment includes analyzing the number of communication rounds and the data transfer costs for each round between the central server and the client devices. Additionally, computational efficiency is measured by considering the number of floating-point operations (FLOPs) and the overall execution time. FL distributes the training across multiple client devices, requiring synchronization and management, which introduces additional system complexity. The primary sources of overhead in FL include communication, computation, and storage, while PFL introduces further overhead due to the need for managing additional model variants and adaptive optimization, which increase both local and global computational demands. Assuming there are K = 20 clients with a participation ratio of *r* = 60%, the time required for each round of execution is calculated by considering the proportion of time allocated to communication (both downloading and uploading), local training, and server operations (which include model aggregation and distribution).

A comparison of execution times for various algorithms, illustrated in Fig. 6, reveals that LG-FedAvg is the fastest, as clients only need to communicate shared aggregated parameters with the server, experiences the longest execution times, as participating clients require additional downloads for other models to refine their local models. Similarly, pFedMe incurs slightly longer execution times than other methods due to the additional computational demands of concurrently training personalized local models. RMPFD’s execution time closely aligns with that of FedProx and FedAvg. While RMPFD introduces a slight additional overhead per round, it substantially accelerates convergence, reaching target accuracy in fewer communication rounds. For a given accuracy level, the total execution time is calculated by multiplying the per-round execution time by the number of rounds required. Our findings highlight that most of the execution time per round is allocated to local training rather than communication or server-side operations. This sugwagests that execution time is primarily shaped by the computational capacity of edge devices and the complexity of local model training.

Table 6 examines the communication cost per client, detailing the data exchanged between each client and the server during a single round of training. Communication cost is calculated as the total number of bytes downloaded and uploaded per round. In standard FL methods such as FedAvg and FedProx, each participating client begins by downloading the global model from the server and, at the end of the round, uploads its updated local model back to the server. Personalized approaches like pFedSD and pFedMe maintain similar communication patterns without introducing additional overhead. However, FedPer and LG-FedAvg reduce communication costs by only transmitting specific model portions: FedPer exchanges the “body” of the model, while LG-FedAvg transmits only the “head.” In the RMPFD configuration, only the last two layers (the head) are exchanged, further reducing communication overhead compared to LG-FedAvg. This selective model-sharing approach significantly decreases data transfer requirements per client, contributing to a more efficient communication model. Each algorithm’s communication cost is thus defined by the amount of data exchanged per round, with pFedSD and pFedMe keeping costs minimal, while FedFomo incurs additional overhead as clients must download supplementary data.

The communication cost is determined by the total data transferred between the client and the server in a single round. Each algorithm carries its communication costs; pFedSD and pFedMe do not add extra communication overhead, while FedFomo introduces additional communication costs because clients need to download extra data. The computational cost is determined by the number of FLOPs required for local training for each client in a single round. Different algorithms have varying computational costs, with RMPFD showing a 5.6% increase compared to FedAvg. Computational cost, measured in FLOPs, reflects the processing demand of local training on each client per round. While RMPFD exhibits a modest increase in computational load (7.3% more than FedAvg), this added cost per round is effectively offset by RMPFD’s ability to converge to the target accuracy in fewer rounds, balancing the computational demand with reduced overall training time.

Table 7 analyzes the computational cost associated with local training for each client per round, measured in FLOPs. These costs are calculated using the FLOPs-counting tool, pytorch-OpCounter3, where the baseline computational requirement for FedAvg is recorded at 904.25 gigabytes. LG-FedAvg and FedPer incur comparable computational costs to FedAvg, as their architecture does not require additional local processing. However, algorithms such as FedProx and pFedMe introduce a marginal increase in computational demand due to the calculation of regularization terms, which serve to enhance model robustness under data heterogeneity. FedFomo, by contrast, requires extra computation for determining a weight vector to aggregate client models before local training begins.

**Fig. 10:**
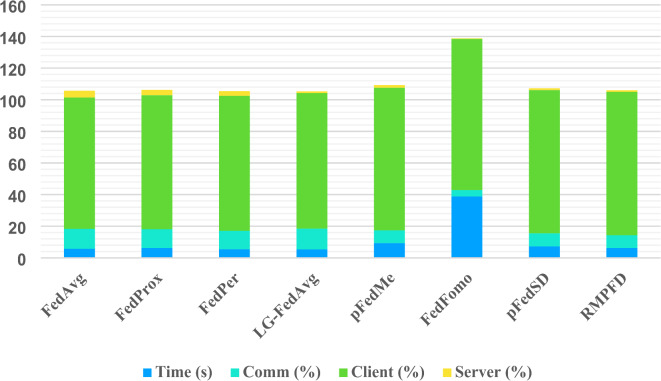
Average Execution Time per Round on CIFAR- 100: Breakdown of Communication, Local Training, and Server Aggregation Overheads.

The average execution time for each round on the CIFAR- 100 dataset, along with a detailed analysis of the overheads associated with communication, local training, and server aggregation shown in Fig. 10. In the proposed RMPFD approach, meta-learning and knowledge distillation techniques are employed to generate soft targets for training in two ways: either by retaining the previous predictions of the personalized model or by recalculating predictions during the local training process. In the primary method, storing previous predictions avoids adding any further computational cost.

**Table 7:** Communication cost and computational cost per round on CIFAR- 100.

Algorithm	Communication overhead (MB)	Computational load (FLOPs)
FedAvg	1.88	904.25G
FedProx	1.88	906.15G
FedPer	1.88	904.25G
LG-Fedavg	0.05	904.25G
pFedMe	1.88	907.35G
FedFomo	6.42	960.51G
pFedSD	1.88	965.43G
RMPFD	1.88	965.43G

## Conclusion and future work

This study presents a novel framework for collaborative personalized federated learning, RMPFD, designed to enhance knowledge transfer by adapting a global model to account for statistical variations in decentralized edge environments. RMPFD effectively combines high-entropy and tailored local models, addressing challenges related to model heterogeneity while ensuring gradient privacy. Extensive evaluations on Fashion-MNIST, CIFAR- 10, CIFAR- 100 and Enron email datasets demonstrate that RMPFD significantly enhances convergence speed and minimizes data loss compared to existing baselines, including FedProx and FedPer across all examined datasets. The empirical results show that after just five rounds, the data loss is reduced to only 0.5% on the CIFAR- 10 dataset, and it converges within 20 rounds, decreasing data loss to 0.1% on the CIFAR- 100. A key contribution is its capability to classify and tailor the global model according to each client’s unique data distribution, facilitating a highly personalized and efficient federated learning process. Furthermore, RMPFD demonstrates robust resilience against gradient leakage attacks and offers enhanced privacy assurances. Moreover, it leverages historically personalized models through knowledge distillation to effectively balance the trade-off between personalization and generalization.

Future work could broaden the scope of this study by evaluating the framework’s performance with a diverse range of client data exhibiting complex heterogeneity. Further investigations might also focus on developing advanced aggregation capable of accommodating varied client data, particularly in healthcare and business applications, where privacy-preserving personalized mutual learning is crucial.

## Data Availability

The datasets utilized and/or analyzed in the current study can be accessed via the following links. The MNIST dataset is available in the LEAF benchmark at https://leaf.cmu.edu/. The CIFAR- 10 dataset is available at https://www.cs.toronto.edu/~kriz/cifar.html. The Enron Email dataset is available at https://www.cs.cmu.edu/~./enron/.

## References

[1] Yang, Q., Liu, Y., Chen, T. & Tong, Y. Federated machine learning: Concept and applications. *ACM Trans. Intell. Syst. Technol.* **10**(2), 1–19. 10.1145/3298981 (2019).

[2] Liu, J. et al. From distributed machine learning to federated learning: A survey. *Knowl. Inf. Syst.* **64**(4), 885–917. 10.1007/s10115-022-01664-x (2022).

[3] Dai, S. & Meng, F. Addressing modern and practical challenges in machine learning: A survey of online federated and transfer learning. *Appl. Intell.* **53**(9), 11045–11072. 10.1007/s10489-022-04065-3 (2023).

[4] Richtárik, P. & Takáč, M. Distributed coordinate descent method for learning with big data. *J. Mach. Learn. Res.* **17**(75), 1–15 (2016).

[5] Shen, S., Zhu, T., Wu, D., Wang, W. & Zhou, W. From distributed machine learning to federated learning: In the view of data privacy and security. *Concurr. Comput.: Practice Exp.*10.1002/cpe.6002 (2022).

[6] Mozib, M. Distributed deep learning based framework to optimize real-time offloading in mobile edge computing networks. *Int. J. Sci. Res.* **12**(6), 1812–1827. 10.21275/sr23603125305 (2023).

[7] Liao, Y. et al. Decentralized federated learning with adaptive configuration for heterogeneous participants. *IEEE Trans. Mob. Comput.* **23**(6), 7453–7469. 10.1109/TMC.2023.3335403 (2024).

[8] Brendan McMahan, H., Moore, E., Ramage, D., Hampson, S. & Agüera y Arcas, B. Communication-efficient learning of deep networks from decentralized data. In *Proceedings of the 20th International Conference on Artificial Intelligence and Statistics, AISTATS 2017*, (2017).

[9] Tran, A. T., Luong, T. D., Karnjana, J. & Huynh, V. N. An efficient approach for privacy preserving decentralized deep learning models based on secure multi-party computation. *Neurocomputing* **422**, 245–262. 10.1016/j.neucom.2020.10.014 (2021).

[10] Qi, P. et al. FedBKD: Heterogenous federated learning via bidirectional knowledge distillation for modulation classification in IoT-edge system. *IEEE J. Selected Topics Signal Process.* **17**(1), 189–204. 10.1109/JSTSP.2022.3224597 (2023).

[11] Luo, B. et al. Optimization design for federated learning in heterogeneous 6G networks. *IEEE Netw.* **37**(2), 38–43. 10.1109/MNET.006.2200437 (2023).

[12] Nishio, T. & Yonetani, R. Client selection for federated learning with heterogeneous resources in mobile edge. *IEEE Int. Conf. Commun.*10.1109/ICC.2019.8761315 (2019).

[13] Huang, Z. A. et al. Federated multi-task learning for joint diagnosis of multiple mental disorders on MRI scans. *IEEE Trans. Biomed. Eng.* **70**(4), 1137–1149. 10.1109/TBME.2022.3210940 (2023). 36178988 10.1109/TBME.2022.3210940

[14] Gad, G. & Fadlullah, Z. Federated learning via augmented knowledge distillation for heterogenous deep human activity recognition systems. *Sensors* **23**(1), 6. 10.3390/s23010006 (2023). 10.3390/s23010006 PMC9823596 36616609

[15] Reddi, S. J. et al. Adaptive federated optimization. In *ICLR 2021 - 9th International Conference on Learning Representations*, (2021).

[16] Li, T., Sahu, A. K., Talwalkar, A. & Smith, V. Federated learning: Challenges, methods, and future directions. *IEEE Signal Process. Mag.* **37**(3), 50–60. 10.1109/MSP.2020.2975749 (2020).

[17] Guendouzi, B. S., Ouchani, S., EL Assaad, H. & EL Zaher, M. A systematic review of federated learning: Challenges, aggregation methods, and development tools. *J. Network Comput. Appl.* **220**, 103714. 10.1016/j.jnca.2023.103714 (2023).

[18] Guo, C., Jia, J., Jie, Y., Liu, C. Z. & Choo, K. K. R. Enabling secure cross-modal retrieval over encrypted heterogeneous IoT databases with collective matrix factorization. *IEEE Internet Things J.* **7**(4), 3104–3113. 10.1109/JIOT.2020.2964412 (2020).

[19] Wang, Z., Peng, C., He, X. & Tan, W. Wasserstein distance-based deep leakage from gradients. *Entropy* **25**(5), 810. 10.3390/e25050810 (2023). 37238565 10.3390/e25050810 PMC10217429

[20] Phong, L. T., Aono, Y., Hayashi, T., Wang, L. & Moriai, S. Privacy-preserving deep learning via additively homomorphic encryption. *IEEE Trans. Inform. Forensics Secur.* **13**(5), 1333–1345. 10.1109/TIFS.2017.2787987 (2018).

[21] Brendan McMahan, H., Moore, E., Ramage, D., Hampson, S. & Agüera y Arcas, B. Federated learning of deep networks using model averaging. In *Proceedings of the 20th International conference on artificial intelligence and statistics, AISTATS 2017*, (2017).

[22] Karimireddy, S. P., Kale, S., Mohri, M., Reddi, S. J., Stich, S. U. & Suresh, A. T. SCAFFOLD: Stochastic controlled averaging for federated learning. In *37th international conference on machine learning, ICML 2020*, (2020).

[23] Rakhlin, A., Shamir, O. & Sridharan, K. Making gradient descent optimal for strongly convex stochastic optimization. In *Proceedings of the 29th international conference on machine learning, ICML 2012*, (2012).

[24] Wang, L., Wang, W. & Li, B. CMFL: Mitigating communication overhead for federated learning. *Proc. – Int. Conf. Distribut. Comput. Syst.*10.1109/ICDCS.2019.00099 (2019).

[25] Wen, D., Jeon, K. J. & Huang, K. Federated dropout - A simple approach for enabling federated learning on resource constrained devices. *IEEE Wireless Commun. Lett.* **11**(5), 923–927. 10.1109/LWC.2022.3149783 (2022).

[26] Kaushal, V. & Sharma, S. Weighted FedCOM: A communication efficient approach to federated learning. *Evolv. Syst.* **16**(1), 27 (2025).

[27] Sun, H. et al. FedDGA: Federated multi-task learning based on dynamic guided attention. *IEEE Trans. Artif. Intell.*10.1109/TAI.2024.3350538 (2024).

[28] Liu, W. et al. Federated meta reinforcement learning for personalized tasks. *Tsinghua Sci. Technol.* **29**(3), 911–926. 10.26599/TST.2023.9010066 (2023).

[29] Fallah, A., Mokhtari, A. & Ozdaglar, A. Personalized federated learning with theoretical guarantees: A model-agnostic meta-learning approach. *Adv. Neural Inf. Process. Syst.* **33**, 3557–3568 (2020).

[30] Shaik, T. et al. Clustered FedStack: Intermediate global models with bayesian information criterion. *Pattern Recogn. Lett.* **177**, 121–127. 10.1016/j.patrec.2023.12.004 (2024).

[31] Jie, Z., Chen, S., Lai, J., Arif, M. & He, Z. Personalized federated recommendation system with historical parameter clustering. *J. Ambient Intell. Hum. Comput.* **14**(8), 10555–10565. 10.1007/s12652-022-03709-z (2023).

[32] Liang, B., Cai, J. & Yang, H. A new cell group clustering algorithm based on validation & correction mechanism. *Expert Syst. Appl.* **193**, 116410. 10.1016/j.eswa.2021.116410 (2022).

[33] Zhang, M., Sapra, K., Fidler, S., Yeung, S. & Alvarez, J. M. Personalized federated learning with first order model optimization. In *ICLR 2021 - 9th international conference on learning representations*, (2021).

[34] Arivazhagan, M. G., Aggarwal, V., Singh, A. K. & Choudhary, S. Federated learning with personalization layers, (2019).

[35] Huang, Y. et al., Personalized cross-silo federated learning on non-IID data. In *35th AAAI conference on artificial intelligence, AAAI 2021*, (2021). 10.1609/aaai.v35i9.16960.

[36] Zhao, Z. et al. Ensemble federated learning with non-IID data in wireless networks. *IEEE Trans. Wirel. Commun.*10.1109/TWC.2023.3309376 (2023).

[37] Tsankova, P. & Momcheva, G. Sentiment detection with FedMD: Federated learning via model distillation. In *CEUR Workshop Proceedings*, (2020).

[38] Gou, J., Yu, B., Maybank, S. J. & Tao, D. Knowledge distillation: A survey. *Int. J. Comput. Vis.* **129**(6), 1789–1819. 10.1007/s11263-021-01453-z (2021).

[39] Li, X., Chen, B. & Lu, W. FedDKD: Federated learning with decentralized knowledge distillation. *Appl. Intell.* **53**(15), 18547–18563. 10.1007/s10489-022-04431-1 (2023).

[40] Cui, Z., Du, L., Wang, P., Cai, X. & Zhang, W. Malicious code detection based on CNNs and multi-objective algorithm. *J. Parallel Distrib. Comput.* **129**, 50–58. 10.1016/j.jpdc.2019.03.010 (2019).

[41] Wang, X., Liu, Z. & Huang, B. Robust and privacy-preserving federated learning scheme based on ciphertext-selected users. *Comput. Netw.* **259**, 111072 (2025).

[42] Wei, Q. EVFL-DCs: Enhancing verifiability of federated learning by double commitments based on blockchain. *Comput. Netw.*, (2025).

[43] Huynh-The, T. et al. Blockchain for the metaverse: A review. *Fut. Generat. Comput. Syst.* **143**, 401–419. 10.1016/j.future.2023.02.008 (2023).

[44] Guo, J. et al. TFL-DT: A trust evaluation scheme for federated learning in digital twin for mobile networks. *IEEE J. Selected Areas Commun.* **41**(11), 3548–3560. 10.1109/JSAC.2023.3310094 (2023).

[45] Ullah, F. et al. Enhancing brain tumor segmentation accuracy through scalable federated learning with advanced data privacy and security measures. *Mathematics* **11**(19), 4189. 10.3390/math11194189 (2023).

[46] Tan, A. Z., Yu, H., Cui, L. & Yang, Q. Towards personalized federated learning. *IEEE Trans. Neural Netw. Learn. Syst.* **34**(12), 9587–9603. 10.1109/TNNLS.2022.3160699 (2023). 35344498 10.1109/TNNLS.2022.3160699

[47] Pentelas, A., De Vleeschauwer, D., Chang, C. Y., De Schepper, K. & Papadimitriou, P. Deep multi-agent reinforcement learning with minimal cross-agent communication for SFC partitioning. *IEEE Access* **11**, 40384–40398. 10.1109/ACCESS.2023.3269576 (2023).

[48] Xu, Y. et al. Federated learning with client selection and gradient compression in heterogeneous edge systems. *IEEE Trans. Mob. Comput.*10.1109/TMC.2023.3309497 (2023).

[49] Zhang, J., Tao, Z., Zhang, S., Qiao, Z. & Guo, K. Soft hybrid knowledge distillation against deep neural networks. *Neurocomputing* **570**, 127142. 10.1016/j.neucom.2023.127142 (2024).

[50] Dong, F. et al. PADP-FedMeta: A personalized and adaptive differentially private federated meta learning mechanism for AIoT. *J. Syst. Architect.* **134**, 102754. 10.1016/j.sysarc.2022.102754 (2023).

[51] Zhu, Z., Hong, J. & Zhou, J. Data-free knowledge distillation for heterogeneous federated learning. In *Proceedings of Machine Learning Research*, (2021). PMC9036494 35480385

[52] Zhang, T., Zhu, T., Gao, K., Zhou, W. & Yu, P. S. Balancing learning model privacy, fairness, and accuracy with early stopping criteria, (2023). 10.1109/TNNLS.2021.3129592. 10.1109/TNNLS.2021.3129592 34878980

[53] Hao, W., Hao, W., Wang, J., Yang, H. & Li, F. A novel method for Jinnan cattle individual classification based on deep mutual learning. *Syst. Sci. Control Eng.* **11**(1), 2207587. 10.1080/21642583.2023.2207587 (2023).

[54] Kaur, A., Kaushal, C., Sandhu, J. K., Damaševičius, R. & Thakur, N. Histopathological image diagnosis for breast cancer diagnosis based on deep mutual learning. *Diagnostics* **14**(1), 95. 10.3390/diagnostics14010095 (2024). 10.3390/diagnostics14010095 PMC10795733 38201406

[55] Li, J., Yang, C., Ye, G. & Nguyen, Q. V. H. Graph neural networks with deep mutual learning for designing multi-modal recommendation systems. *Inf. Sci. (N Y)* **654**, 119815. 10.1016/j.ins.2023.119815 (2024).

[56] Miao, Y. et al. Efficient and secure federated learning against backdoor attacks. *IEEE Trans. Dependable Secure Comput.*10.1109/TDSC.2024.3354736 (2024).

[57] Jiao, S., Cai, L., Wang, X., Cheng, K. & Gao, X. A differential privacy federated learning scheme based on adaptive Gaussian noise. *Comput. Model. Eng. Sci.*, **138**(2), (2024). 10.32604/cmes.2023.030512.

[58] He, Z., Wang, L. & Cai, Z. Clustered federated learning with adaptive local differential privacy on heterogeneous IoT data. *IEEE Internet Things J.* **11**(1), 137–146. 10.1109/JIOT.2023.3299947 (2024).

[59] Hijazi, N. M., Aloqaily, M., Guizani, M., Ouni, B. & Karray, F. Secure federated learning with fully homomorphic encryption for IoT communications. *IEEE Internet Things J.* **11**(3), 4289–4300. 10.1109/JIOT.2023.3302065 (2024).

[60] Ling, J., Zheng, J. & Chen, J. Efficient federated learning privacy preservation method with heterogeneous differential privacy. *Comput. Secur.* **139**, 103715. 10.1016/j.cose.2024.103715 (2024).

[61] Thakur, A., Sharma, P. & Clifton, D. A. Dynamic neural graphs based federated reptile for semi-supervised multi-tasking in healthcare applications. *IEEE J. Biomed. Health Inform.* **26**(4), 1761–1772. 10.1109/JBHI.2021.3134835 (2022). 34898443 10.1109/JBHI.2021.3134835 PMC7615588

[62] Kapsecker, M., Nugraha, D. N., Weinhuber, C., Lane, N. & Jonas, S. M. Federated learning with swift: An extension of flower and performance evaluation. *SoftwareX*, **24**, (2023). 10.1016/j.softx.2023.101533.

[63] Krizhevsky, A., Nair, V. & Hinton, G. CIFAR-10 and CIFAR-100 datasets, (2009).

[64] Krizhevsky, A. Learning multiple layers of features from tiny images. *Science Department, University of Toronto, Tech. *(2009), 10.1.1.222.9220.

[65] Li, Q., Diao, Y., Chen, Q. & He, B. Federated learning on Non-IID data silos: An experimental study. *Proc. – Int. Conf. Data Eng.*10.1109/ICDE53745.2022.00077 (2022).

[66] Zhang, S., Choromanska, A. & LeCun, Y. Deep learning with elastic averaging SGD. In *3rd International Conference on Learning Representations, ICLR 2015 - Workshop Track Proceedings*, (2015).

[67] Li, X., Yang, W., Zhang, Z., Huang, K. & Wang, S. On the convergence of Fedavg on non-IID data. In *8th International Conference on Learning Representations, ICLR 2020*, (2020).

[68] Dinh, C. T., Tran, N. & Nguyen, J. Personalized federated learning with moreau envelopes. *Adv. Neural Inform. Process. Syst.* **33**, 21394–21405 (2020).

